# Bisecting GlcNAc modification reverses the chemoresistance via attenuating the function of P-gp

**DOI:** 10.7150/thno.93879

**Published:** 2024-08-19

**Authors:** Zengqi Tan, Lulu Ning, Lin Cao, Yue Zhou, Jing Li, Yunyun Yang, Shuai Lin, Xueting Ren, Xiaobo Xue, Huafeng Kang, Xiang Li, Feng Guan

**Affiliations:** 1 Institute of Hematology, Provincial Key Laboratory of Biotechnology, School of Medicine, Northwest University, Xi'an, Shaanxi, 710069, P.R. China.; 2 College of Bioresources Chemical and Materials Engineering, Shaanxi University of Science & Technology, Xi'an, Shaanxi, 710069, P.R. China.; 3 Key Laboratory of Resource Biology and Biotechnology in Western China, Ministry of Education, Provincial Key Laboratory of Biotechnology, College of Life Sciences, Northwest University, Xi'an, Shaanxi, 710069, P.R. China.; 4 Department of Oncology, The Second Affiliated Hospital of Xi'an Jiaotong University, Xi'an, Shaanxi, 710069, P.R. China.

**Keywords:** Bisecting GlcNAc, P-glycoprotein, MGAT3, Chemoresistance, Microvesicles

## Abstract

**Rationale:** Chemoresistance is a key factor contributing to the failure of anti-breast cancer chemotherapy. Although abnormal glycosylation is closely correlated with breast cancer progression, the function of glycoconjugates in chemoresistance remains poorly understood.

**Methods:** Levels and regulatory roles of bisecting N-acetylglucosamine (GlcNAc) in chemoresistant breast cancer cells were determined in vitro and in vivo. Glycoproteomics guided identification of site-specific bisecting GlcNAc on P-glycoprotein (P-gp). Co-immunoprecipitation coupled mass spectrometry (Co-IP-MS) and proximity labelling MS identified the interactome of P-gp, and the biological function of site-specific bisecting GlcNAc was investigated by site/truncation mutation and structural simulations.

**Results:** Bisecting GlcNAc levels were reduced in chemoresistant breast cancer cells, accompanied by an enhanced expression of P-gp. Enhanced bisecting GlcNAc effectively reversed chemoresistance. Mechanical study revealed that bisecting GlcNAc impaired the association between Ezrin and P-gp, leading to a decreased expression of membrane P-gp. Bisecting GlcNAc suppressed VPS4A-mediated P-gp recruitment into microvesicles, and chemoresistance transmission. Structural dynamics analysis suggested that bisecting GlcNAc at Asn494 introduced structural constraints that rigidified the conformation and suppressed the activity of P-gp.

**Conclusion:** Our findings highlight the crucial role of bisecting GlcNAc in chemoresistance and suggest the possibility of reversing chemoresistance by modulating the specific glycosylation in breast cancer therapy.

## Introduction

Breast cancer (BC) is the most commonly diagnosed malignancy among women worldwide 1. While chemotherapy is initially effective in treating BC, a significant number of patients eventually experience relapse with chemotherapy-resistant tumors 2. Therefore, the development of chemoresistance poses a major obstacle to successful chemotherapy of BC. Among the underlying mechanisms of chemoresistance, P-glycoprotein (P-gp, also referred to as MDR1), one of the ATP-binding cassette (ABC) family of membrane transport proteins, is one major cause of chemoresistance. P-gp expression is detected in a significant percentage of BC patients 3,4, and is commonly elevated in response to chemotherapeutic drugs (particularly P-gp substrates), correlating with poor clinical outcomes 3. Therefore, P-gp inhibitors were extensively introduced for the sensitization of chemotherapeutic drugs, and several inhibitors were investigated in different phases of BC clinical trials 5.

P-gp is a membrane glycoprotein with multiple glycosylation sites 6. Glycosylation, a crucial post-translational modification, affects the folding, trafficking, stability, and activity of glycoproteins 7. Abnormal glycosylation has been associated with cancer initiation, progression, angiogenesis, invasion, metastasis, and chemoresistance. For instance, increased O-GlcNAcylation levels results in chemoresistance in BC cells through activating survival signaling 8. B4GALT1, a glycosyltransferase, is mainly responsible for the biosynthesis of glycan structure Galβ1,4-GlcNAc, is up-regulated in gemcitabine-resistant pancreatic cancer patient-derived organoids and chemoresistant cell lines, and depletion of B4GALT1 reverses the chemoresistance to gemcitabine 9. The changes in P-gp glycosylation between drug-sensitive and resistant gastric cancer cells have a significant impact on its function in gastric cancer multidrug resistance 10. While it is confirmed that N-glycosylation helps maintain the localization of P-gp on the cell membrane 11, the exact modulation of glycosylation on P-gp is not yet clear.

Bisecting GlcNAc, a specific type of N-glycosylation characterized by a β1,4-linked GlcNAc attached to a core β-mannose residue and catalyzed by N-acetylglucosaminyltransferase MGAT3, regulates processing and elongation of N-glycans on proteins 12. Bisecting GlcNAc plays a role in various biological functions of glycoproteins involved in cell adhesion, migration, growth, and differentiation 13,14. Our group has shown that bisecting GlcNAc is down-regulated in BC, and the overexpression of MGAT3 increases bisecting GlcNAc levels on EGFR, and suppresses EGFR/Erk signaling 15. Additionally, we have found that high levels of bisecting GlcNAc suppress the metastasis of recipient cells induced by small extracellular vesicles derived from BC cells 16.

In our current study, we observed a decreased level of bisecting GlcNAc in chemoresistant BC cells and tissues, accompanied by an increased P-gp expression. We also discovered that P-gp can be modified by bisecting GlcNAc. Consequently, our primary objective is to delve into the molecular mechanisms that govern how bisecting GlcNAc influences the expression and functionality of P-gp.

## Methods

### Patient samples

Serum of normal subjects, chemoresistant and chemosensitive BC patients, and tissues from chemoresistant and chemosensitive BC patients were obtained from the Second Affiliated Hospital of Xi'an Jiaotong University. Written informed consent was obtained from all patients in accordance with the Declaration of Helsinki. Experiments using human tissues were approved by the Research Ethics Committee of Northwest University (approval number: 220224018, 24 February 2022). BC patients who have received neoadjuvant chemotherapy (NACT) were divided into chemoresistant group (grade 1 to 2) and chemosensitive group (grade 4 to 5) according to the Miller-Payne grading system. Tissues from chemoresistance and chemosensitive BC patients post- and pre- NACT, and serum from chemoresistance and chemosensitive BC patients post-NACT were obtained.

### Cell lines and cell culture

Human BC cell lines (MCF7, MDA-MB-231 and BT-549) and HEK293T were purchased from the Cell Bank at the Chinese Academic of Science. Chemoresistant MCF7 cells were developed by stepwise selection in increasing doses of doxorubicin (DOX; MedChemExpress) or paclitaxel (PTX, MedChemExpress) as previously described 17,18, termed MCF7^DOX^ and MCF7^PTX^, respectively. MCF7, MDA-MB-231, MCF7^DOX^, MCF7^PTX^ and HEK293T cells were cultured in Dulbecco's Modified Eagle's Medium (Gibco). BT549 cells were cultured in RPMI 1640 (Gibco). All medium was supplemented with 10% FBS (Biological Industries), 100 UI/mL penicillin, and 100 μg/mL streptomycin (Gibco).

### Stable transfection of MGAT3

MGAT3 was amplified via PCR and cloned into the lentiviral vector, pLVX-AcGFP-N1 (Takara). The vector was packaged in HEK293T cells using Lipofectamine 2000 regent (Thermofisher Scientific), together with pMD2.G (Addgene plasmid # 12259; RRID: Addgene_12259) and psPAX2 (Addgene plasmid # 12260; RRID: Addgene_12260). MDA-MB-231, MCF7 and MCF7^DOX^ cells were infected with above lentivirus. Cells stably transfected with MGAT3 were screened with puromycin (Beyotime Institute of Biotechnology), and confirmed by western blotting analysis.

### Total protein extraction

Cells were washed with ice-cold 1×PBS (0.01 M phosphate buffer containing 0.15 M NaCl, pH7.4), and added with appropriate amount of RIPA buffer (25 mM Tris-HCl pH7.6, 1% Triton X-100, 1% sodium deoxycholate, 0.1% SDS, 150 mM NaCl, 5 mM EDTA) containing protease inhibitor (NCM Biotech), and lysed on ice for 30 min. Lysates were centrifugated at 14,000 × g for 15 min at 4°C, and supernatant was collected. Protein content was determined by BCA assay (Beyotime).

### Western blotting and lectin blotting

Proteins were loaded and separated by SDS-PAGE, and electroblotted onto polyvinylidene difluoride (PVDF) membranes (Bio-Rad). Membranes were blocked with 5% (w/v) BSA (Beyotime) in TBST (20 mM Tris, pH 7.5, 150 mM NaCl, 0.1% Tween-20) for 1 h at 37°C, probed with primary antibodies or biotin-conjugated PHA-E (Vector Laboratories Cat# B-1125, RRID: AB_2336650) overnight at 4°C, and incubated with appropriate HRP-conjugated secondary antibody (Beyotime) or streptavidin (Vector Laboratories Cat# PK-4000, RRID: AB_2336818). Bands were visualized by enhanced chemiluminescence (ECL; Tanon Science and Technology).

### Real-time PCR (qRT-PCR)

Total RNA was isolated using Total RNA Extraction Reagent (Vazyme), and cDNAs were synthesized using a HiScript II 1st Strand cDNA Synthesis Kit (Vazyme). Quantitative real-time PCR was performed using a CFX96 Touch PCR Detection System (Bio-Rad) with a ChamQ SYBR qPCR Master Mix kit (Vazyme). Gene expression was quantified using the 2^-ΔΔCT^ method.

### Enzyme-linked immunosorbent assay (ELISA)

ELISA plates (Jet Biofil) were coated with patient serum samples, incubated with shaking (200 rpm) for 2 h at 37°C, blocked with 3% BSA in PBS for 2 h at room temperature (RT), and rinsed with 0.1% Tween-20 in PBS (TPBS). Plates were incubated with biotinylated PHA-E (2 μg/mL) for 30 min at RT, rinsed with TPBS, incubated with HRP-streptavidin for 30 min at RT, and rinsed with TPBS. Wells were added with 3,3',5,5'-tetramethyl benzidine (TMB, Beyotime), and the reaction was terminated with acid stop solution (2 M sulfuric acid). Optical density at 450 nm was determined by plate reader.

### Immunofluorescence staining

Cells cultured on glass coverslips were rinsed with ice-cold PBS, immobilized with 4% fresh paraformaldehyde for 15 min at RT, blocked with 5% BSA for 1 h at 37°C, incubated with fluorescence-labeled lectin or primary antibody overnight at 4°C, rinsed with PBS, incubated with fluorescence-labeled secondary antibody with DAPI for 30 min at RT, and rinsed with PBS. Images were captured by confocal fluorescence microscopy (TCS SP8, Leica). Colocalization between proteins of interests was selected and quantified by Image J software (version 1.54J) using the colocalization finder plug-in.

### Duolink in situ proximity ligation assay (PLA)

PLA was conducted using Duolink® In Situ Red Starter Kit Mouse/Rabbit according to the manufacturer's protocol (Sigma-Aldrich). Briefly, cells were immobilized with 4% fresh paraformaldehyde, permeabilized with 0.2% Triton X-100, blocked with blocking solution, incubated with primary antibodies overnight at 4°C, probed with secondary antibodies conjugated with oligonucleotides for 60 min at 37°C, followed by incubation with ligation-ligase solution and amplification-polymerase solution, respectively. Fluorescence signal was captured by confocal fluorescence microscopy.

### Tissue microarray analysis/immunohistochemistry

Tissue microarray (TMA) / immunohistochemistry was performed as previously described 16. TMA (HBreD140Su03) of BC were from Shanghai Outdo Biotech Co. TMA and paraffin-embedded slides of BC tissues were deparaffinized, rehydrated, and subjected to antigen retrieval by microwave irradiation in 10 mM citrate buffer (10 mM sodium citrate, 0.05% Tween 20, pH 6.0) in a 750-W oven for 30 min at 95-97°C. The slides were blocked with hydrogen peroxide in methanol, incubated with antibodies against MGAT3 (Proteintech Cat# 17869-1-AP, RRID: AB_10597697) or P-gp (Proteintech Cat# 22336-1-AP, RRID: AB_2833023) or biotinylated PHA-E overnight at 4°C, rinsed with PBS, and then incubated with secondary antibody or HRP-streptavidin for 1 h at 37°C, and visualized with DAB reagent (Beyotime). The mean optical density of the staining signal was calculated using ImagePro Plus software (Media Cybernetics, RRID:SCR_016879).

### Immunoprecipitation (IP)

Lysates (500 μg) were added with 2 μg primary antibody for 2 h at 4°C, and incubated with 20 μL Protein A/G Plus-Agarose (Santa Cruz) overnight at 4°C. The mixture was rinsed with ice-cold PBS, denatured with SDS loading buffer, collected by centrifugation, and subjected to SDS-PAGE analysis.

### Cell apoptosis

Cell apoptosis was determined as per manufacturer's instructions. In brief, detached cells (2 × 10^5^) were rinsed with ice-cold PBS, and incubated with binding buffer supplemented with APC-conjugated annexin-V (BioLegend Cat# 831603, RRID: AB_2686933) and 7-AAD (BioLegend) for 15 min at RT in the dark. Cells in early apoptosis (annexin V-positive) and in late apoptosis (annexin V-positive and 7-AAD-positive) were quantified by flow cytometry (Novocyte 2060R; ACEA Biosciences).

### Cell viability

Cells were seeded into 96-well cell culture plates (5000 cells/well), incubated with the CCK8 reagent (Enogene Biotech) for 2 h at 37°C, and the absorbance at 450 nm was measured using a plate reader.

### Identification of proteins with bisecting GlcNAc

Proteins were denatured with 8 M urea in 50 mM NH_4_HCO_3_, reduced with DTT, alkylated with IAA, digested with lysyl endopeptidase (Wako Puro Chemical) and trypsin (Promega) as described previously 16. Glycopeptide enrichment was performed by Oasis MAX column (Waters) using 200 μg of digested peptides. Two-dimensional LC-MS were performed using QE HF-X (Thermofisher Scientific), and glycoproteomics data were analyzed with Glyco-Decipher (v1.0.3) 19.

### IP-based mass spectrometry analysis

Immunoprecipitated complexes were separated by SDS-PAGE, and gel pieces were dehydrated with acetonitrile, reduced with DTT, alkylated with IAA and digested with trypsin. Subsequently, the extracted peptides were analyzed using LC-MS/MS (Thermofisher Scientific).

### DOX efflux assay

DOX efflux assay was performed as previously described 20. Chemoresistant cells were incubated with 100 μM DOX for 24 h at 37°C, rinsed with PBS, incubated with fresh media, collected at indicated time points, and resuspended in ice-cold PBS. The residual DOX in cells were determined by flow cytometry analysis.

### Labeling and isolation newly synthesized protein

Cells (~80% confluent) were rinsed with PBS, incubated with methionine and cysteine free DMEM for 1 h at 37°C in 5% CO_2_, rinsed with PBS, incubated with the medium supplemented with 1 mM azidohomoalanine (AHA) for 1 h at 37°C in 5% CO_2_. Total protein extraction was performed as above described. To conjugate AHA-labeled proteins to biotin, total protein extraction was added with 100 μM TBTA, 1 mM CuSO_4_, 100 μM biotin-alkyne, and 1 mM TCEP, and incubated for 1 h at RT. Labeled proteins were further desalted with size-exclusion spin ultrafiltration unit (10 kDa; Millipore), and further subjected to immunoprecipitation and western blotting.

### Surface biotinylation by sulfo-NHS-LC-biotin

Confluent cells were rinsed with ice-cold PBS and surface-biotinylated with 0.5 mg/mL sulfo-NHS-LC-biotin (MedChemExpress) in PBS for 1 h on ice. After removal of excess NHS-LC-biotin, cells were washed with 50 mM Tris-HCl, pH 7.4, containing 100 mM glycine for 10 min on ice, scraped into RIPA buffer containing protease inhibitor, and centrifuged at 14,000 × g for 15 min at 4°C. Protein content was determined by BCA assay, and membranous protein levels were investigated by immunoprecipitation and western blotting.

### Plasma membrane fraction

Confluent cells were rinsed with ice-cold PBS, and harvested. Isolation of the plasma membrane and its associated proteins was conducted with the Minute Plasma Membrane Protein Isolation Kit (Invent Biotech) according to the manufacturer's instructions. The purification of the fractions was analyzed by SDS-PAGE followed by western blotting with the indicated antibodies.

### Animal studies

All mouse experiments were approved by the Animal Care and Use Committee of Northwest University (approval number: NWU-AWC-20211201M, 4 December 2021). For in vivo tumorigenesis assays, 6- to 8-week-old female BALB/c nude mice were purchased from Beijing Vital River Laboratory Animal Technology (Beijing, China). Suspensions of cells (1 × 10^7^ cells/200 μL) were subcutaneously injected into the flanks of the mice. When tumors approached a size of 100 mm^3^, tumor-bearing mice were treated with 2.5 mg·kg^-1^ DOX and/or 5 mg·kg^-1^ forskolin via intraperitoneal injections twice weekly for a total of four injections. Tumor size was measured every other day for 2 weeks, after which tumors were excised and weighed.

### MVs isolation

Cells were cultured in MVs-free FBS medium for 48 h, and culture supernatants were collected and subjected to successive centrifugation at 500 × g for 10 min, 2000 × g for 20 min, and 14,000 × g for 40 min at 4°C, rinsed with ice-cold PBS, and finally resuspended in PBS.

### Proximity labelling and identification

Annexin A1 and Basu-HA were amplified via PCR, fused and linked to lentiviral overexpression vector pLVX-AcGFP-N1. HEK293T cells were transfected with the constructed plasmid using Lipofectamine 2000 reagent (Thermofisher Scientific), incubated with biotin (50 μM) for 18 h, and confirmed by western blot. Transfectants were lysed with RIPA buffer containing protease inhibitor, and cell lysates were collected by centrifugation. Proximity biotinylated proteins were incubated with streptavidin-magnetic beads (Beyotime) overnight, rinsed with PBS, denatured with urea, reduced with DTT, alkylated with IAM, digested with trypsin, purified with Oasis HLB cartridges, and subjected to LC-MS/MS (Thermofisher Scientific).

### Transmission electron microscopy (TEM)

Purified MVs were applied to carbon-coated 400 mesh grids (Electron Microscopy Sciences) for 5 min, and stained with 2% uranyl acetate for 30 s. Images were obtained by TEM (model H-7650; Hitachi) at 80 kV.

### Structural dynamics analysis

To investigate the effects of bisecting GlcNAc on P-gp structure and function, three systems were simulated: P-gp, P-gp with bisecting GlcNAc or branching GlcNAc. Atomic coordinates of P-glycoprotein were determined by homology modeling method and Modeller 9.16 software program was used to model residues 32 to 1275 of P-gp. GLYCAM-Web server was employed to build models of branching and bisecting GlcNAc attached to nitrogen atom of Asn494. All simulations were carried out using the AMBER 16 software 21 together with the AMBER14SB force field 22. Branching and bisecting GlcNAc were modeled by GLYCAM06-j force field 23.

Each system was solvated in a truncated octahedron box with TIP3P waters 24, in which the minimum distance between the input conformation and the edge of the box was at least 12 Å. 10 chloride ions were added to neutralize the system. All the systems were first energy-minimized with a series of position restraints on the solute. The simulation was continued for 100 ns at 1 bar, which was maintained by isotropic position scaling with a 2 ps relaxation time. Langevin thermostat was used to keep the temperature at 300K 25. The SHAKE algorithm 22 was employed to constrain all the bonds involving hydrogens, allowing for a 2 fs integration step. Electrostatic interactions were treated by the particle mesh Ewald sum method 26 and 12 Å non-bonded cutoff was used for van der Waals (VDW) interactions. Five independent MD simulations, 100 ns long per run, were performed in parallel to assess the effects of different glycosylation patterns on the structure of P-gp.

The CPPTRAJ tool 27 was employed for root-mean-square deviation (RMSD), root-mean-square fluctuation (RMSF) calculations with only Cα atoms considered. The RMSD was calculated with respect to the initial structure for each simulation trajectory and the random coil region was omitted (Thr630-Ser691) which was intrinsically flexible. For the RMSF calculation, the average structure of the last 50 ns was used as the reference structure. The formation of hydrogen bonds was determined with the Donor-Acceptor distance 3 Å and Hydrogen-Donor-Acceptor 30° as criterial. Al the molecular structures were rendered by VMD 1.9.3. 28.

### Data analysis

All experiments were performed at least three times. All data are represented as mean±standard deviation (S.D.). Two-tailed Student's *t*-test was used for comparison of data sets between two groups, and differences with *P*<0.05 were considered statistically significant. Statistical analyses were performed using GraphPad Prism V.7.0 software program GraphPad Prism (RRID:SCR_002798). Notations in figures: *, *P*<0.05; **, *P*<0.01; ***, *P*<0.001.

## Results

### Bisecting GlcNAc levels in chemoresistant BC cells

**Figure 1:**
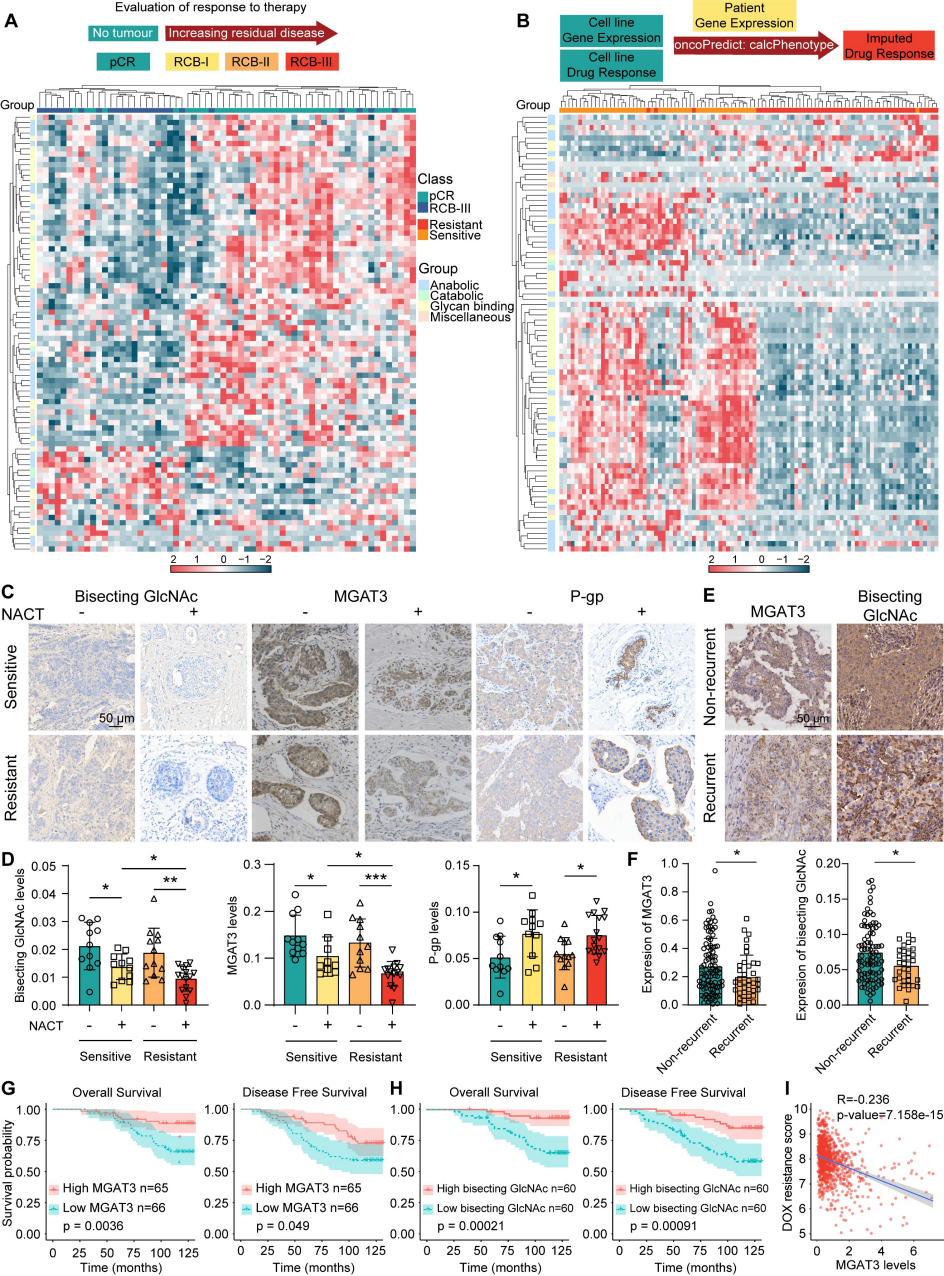
** Bisecting GlcNAc levels in chemoresistant BC cells.** (A) Expression pattern of glycan-related genes in pCR and RCB-III groups 29. (B) Expression pattern of glycan-related genes in imputed DOX resistant and sensitive groups from TCGA datasets by oncoPredict 30. (C&D) Immunohistochemistry of MGAT3, bisecting GlcNAc and P-gp in chemoresistant and chemosensitive BC tissues post- and pre- NACT. (E&F) Differential MGAT3 and bisecting GlcNAc levels of recurrent and non-recurrent BC tissues in TMA. (G&H) Overall and disease-free survival of dichotomized MGAT3 expression (G) and bisecting GlcNAc levels (H) in BC patients by TMA. (I) Correlation analysis of MGAT3 expression and imputed DOX resistance score in BC patients from TCGA database.

To explore the association between chemoresistance and glycans, glycan-related gene expression were profiled using RNA-seq data from BC patients underwent neoadjuvant treatment who had a pathological complete response (pCR) and extensive residual disease (RCB-III) according to residual cancer burden (RCB) classification 29, and using TCGA datasets of BC patients with an imputed response to DOX by oncoPredict 30 (**Fig. 1A&B, Tab. S1**). Specifically, MGAT3 was the significantly altered N-glycan-related gene in chemoresistant BC patients who attained RCB-III or imputed resistance to DOX (**Fig. S1A**).

Similar to chemosensitive BC tissues, chemoresistant tissues exhibited reduced MGAT3 and bisecting GlcNAc levels and elevated P-gp expression following NACT. After receiving NACT, MGAT3 and bisecting GlcNAc levels are reduced in chemoresistant tissues compared to chemosensitive tissues (**Fig. 1C&D**). MGAT3 expression was positively correlated with bisecting GlcNAc levels (**Fig. S1B**). Similarly, bisecting GlcNAc levels in the serum of chemoresistant BC patients were lower (**Fig. S1C**). Tissue microarray (TMA) analysis revealed that MGAT3 and bisecting GlcNAc levels were greatly suppressed in recurrent BC patients (**Fig. 1E&F**), and higher MGAT3 and bisecting GlcNAc levels correlated with improved overall survival and disease-free survival (**Fig. 1G&H**). BC patients with higher MGAT3 mRNA expression exhibited low imputed drug resistance to DOX and PTX 30 (**Fig. S1D&E**). Additionally, among the several tumor-associated glycosyltransferases, MGAT3 mRNA expression were found to be negatively correlated with imputed drug resistance scores in BC patients (**Fig. 1I, S1F-I**). MGAT3 mRNA expression was associated with pCR using simple and multiple logistic regression 29 (**Fig. S1J**). These findings suggest that MGAT3 and its responding bisecting GlcNAc levels are suppressed in BC patients who are resistant to chemotherapy.

### Bisecting GlcNAc partially reverse the chemoresistance in BC cells

**Figure 2:**
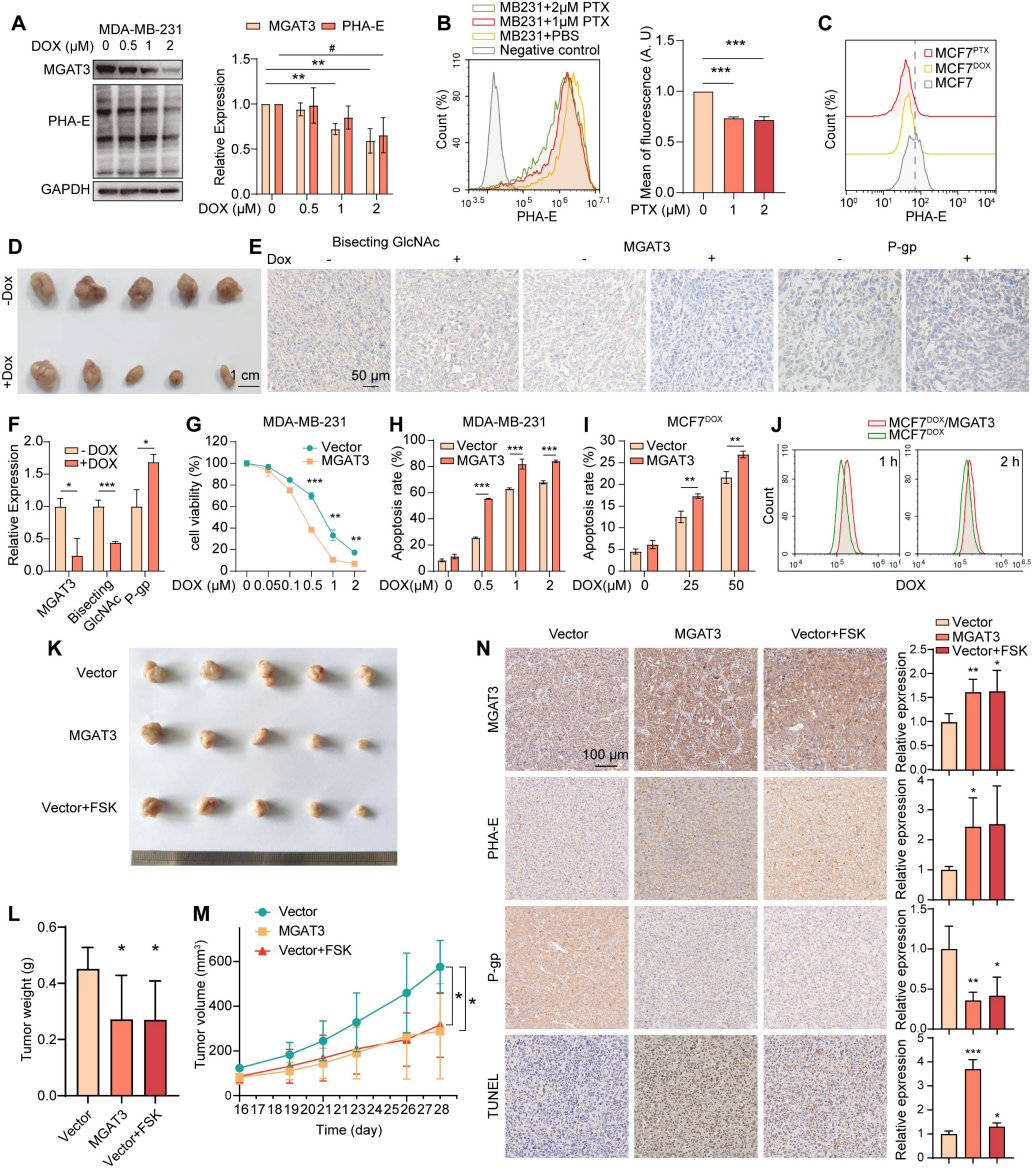
** High bisecting GlcNAc reversed chemoresistance in BC cells.** (A) Bisecting GlcNAc levels and MGAT3 expression in MDA-MB-231 cells treated with different concentration of DOX for 48 h. (B) Bisecting GlcNAc levels in MDA-MB-231 with PTX treatment determined by flow cytometry. (C) Bisecting GlcNAc levels in MCF7, MCF7^DOX^ and MCF7^PTX^ cells determined by flow cytometry. (D) Tumor size of MDA-MB-231 subcutaneous tumor with/ without DOX treatment. (E&F) Immunochemistry of bisecting GlcNAc levels, MGAT3 and P-gp expression in tumors. (G&H) Cell viability (G) and apoptosis (H) assays of parental and MGAT3-overexpressed MDA-MB-231 cells treated with different concentration of DOX for 48 h. (I) Cell apoptosis of parental and MGAT3-overexpressing MCF7^DOX^ cells treated with different concentration of DOX for 48 h. (J) Drug efflux assays of cells. Parental and MGAT3-overexpressed MCF7^DOX^ cells were pre-treated with 100 μM DOX for 24 h, and residual DOX in cells were determined following 1 or 2 hours of drug efflux. (K-M) Tumor size (K), weight (L) and volume (M) of DOX-exposed tumors of MDA-MB-231 or MGAT3 overexpressed MDA-MB-231 cells with/ without forskolin treatment. (N) Histochemistry analysis of MGAT3, bisecting GlcNAc, P-gp and cell apoptosis in tumors.

Bisecting GlcNAc levels and MGAT3 expression were significantly reduced in breast cancer cells MDA-MB-231, MCF7, BT-549 and MCF7^DOX^ response to DOX, PTX or cisplatin in dose- and time-dependent manners (**Fig. 2A&B, S2A-J**), and in MCF7 compared to MCF7^DOX^ and MCF7^PTX^ (**Fig. 2C**). Decreased levels of bisecting GlcNAc and MGAT3 were also observed in DOX-exposed tumor of MDA-MB-231, along with an increased P-gp expression (**Fig. 2D-F**).

To explore the roles of bisecting GlcNAc in BC chemoresistance, we introduced MGAT3 into MDA-MB-231, MCF7 and MCF7^DOX^ cells (termed as MB231/MGAT3, MCF7/MGAT3 and MCF7^DOX^/MGAT3) to enhance bisecting GlcNAc levels (**Fig. S3A**). Upon treatment with DOX or PTX, MB231/MGAT3 cells exhibited decreased cell viability and proliferation, as well as increased apoptosis compared to the parental cells (**Fig. 2G&H, S3B&C**). Similar results were observed in MCF7/MGAT3 and MCF7^DOX^/MGAT3 cells following DOX treatment (**Fig. 2I, S3D-F**), accompanied by weakened drug efflux activity (**Fig. 2J**). On the other hand, treatment of MDA-MB-231 cells with forskolin (FSK), an adenylyl cyclase activator that stimulates MGAT3 expression and increases bisecting GlcNAc levels 31 (**Fig. S3G**), resulted in increased cell apoptosis, as well as reduced cell proliferation (**Fig. S3H**).

In the DOX-treated tumor-bearing nude mice, the introduction of bisecting GlcNAc by either overexpressing MGAT3 or forskolin treatment resulted in a significant reduction in tumor volume and weight (**Fig. 2K-M**), accompanied by an increase in cell apoptosis and a decrease in P-gp expression (**Fig. 2N**).

### MGAT3 degradation in chemoresistant BC cells

**Figure 3:**
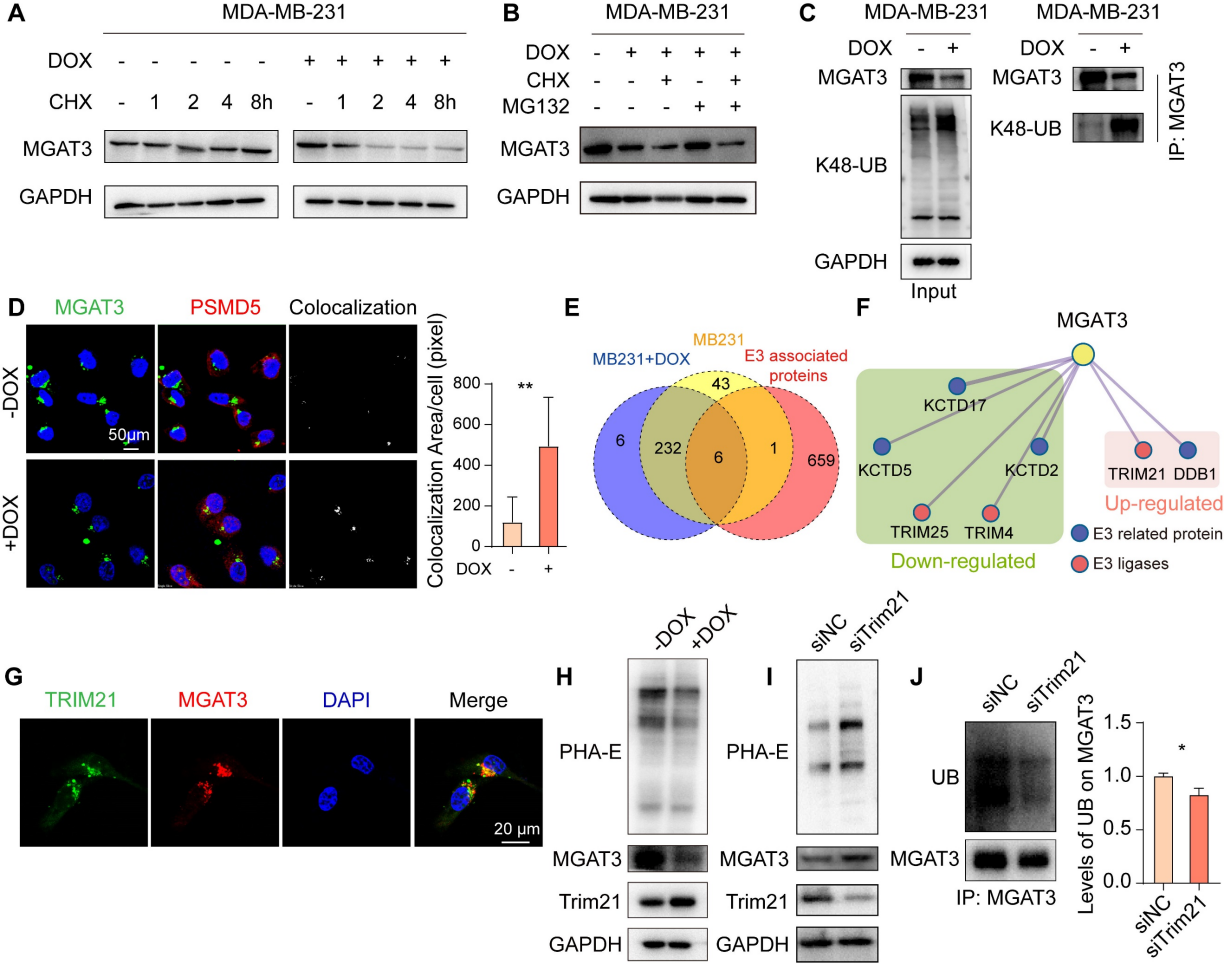
** Trim21 responsible for degradation of MGAT3 in DOX treated BC cells.** (A) Half-time of MGAT3 in CHX-exposed MDA-MB-231 cells with/ without DOX treatment. (B) MGAT3 expression in MDA-MB-231 cells treated with DOX in the presence of MG132 or CHX. (C) K48-linked polyubiquitination levels on MGAT3 in cells treated with/ without DOX. (D) Colocalization between MGAT3 and PSMD5 in cells treated with/ without DOX determined by immunofluorescence. Red: PSMD5, the proteasome marker. Green: MGAT3. Blue: nucleus. Scale bar: 50 mm. Colocalization between MGAT3 and PSMD5 was highlighted, and colocalization area was calculated by Image J software using the colocalization finder plug-in. (E) Venn diagram of MGAT3 associated proteins identified in cells treated with/ without DOX, and known E3 ligases and related proteins. (F) Protein-protein interaction network of E3 ligases and related proteins differentially interacted with MGAT3. (G) Localization of MGAT3 and Trim21 in MDA-MB-231 cells. (H) MGAT3 and Trim21 expression, as well as bisecting GlcNAc levels in cells treated with/ without DOX. (I) Bisecting GlcNAc levels, MGAT3 and Trim21 expression in parental and Trim21 silenced cells. (J) Polyubiquitination levels on MGAT3 in parental and Trim21 silenced cells.

DOX facilitated the degradation of MGAT3 in MDA-MB-231 cells response to the protein synthesis inhibitor, cycloheximide (CHX) (**Fig. 3A**). This degradation of MGAT3 was reversed in DOX-exposed MDA-MB-231 and MCF7 cells by the proteasome inhibitor, MG132, but not by the lysosomal inhibitor, chloroquine (Chol) (**Fig. 3B, S4A-C**). Elevated K48-linked poly-ubiquitination and proteasome localization of MGAT3 were observed in DOX-exposed BC cells (**Fig. 3C&D, S4D**). These data indicate that DOX-induced reduction in MGAT3 is due to the degradation of MGAT3 via the ubiquitin-dependent proteasomal pathway.

To identify the ubiquitin ligase responsible for MGAT3 degradation, we conducted co-immunoprecipitation coupled with mass spectrometry (Co-IP-MS) (**Fig. S4G&H, Tab. S2**). By comparing the proteins identified through Co-IP-MS with known E3 ligases and related proteins, Trim21 was identified to highly interact with MGAT3 in MDA-MB-231 cells response to DOX (**Fig. 3E&F, S4I**), and their co-localization was confirmed by immunofluorescence (**Fig. 3G**). Furthermore, Trim21 expression was increased in response to DOX treatment (**Fig. 3H**), while silencing Trim21 in DOX-exposed BC cells resulted in elevated levels of bisecting GlcNAc and MGAT3 (**Fig. 3I, S4J**), as well as reduced poly-ubiquitination on MGAT3 and increased resistance to chemotherapy (**Fig. 3J, S4K-N**).

### Effect of bisecting GlcNAc on localization and degradation of P-gp

**Figure 4:**
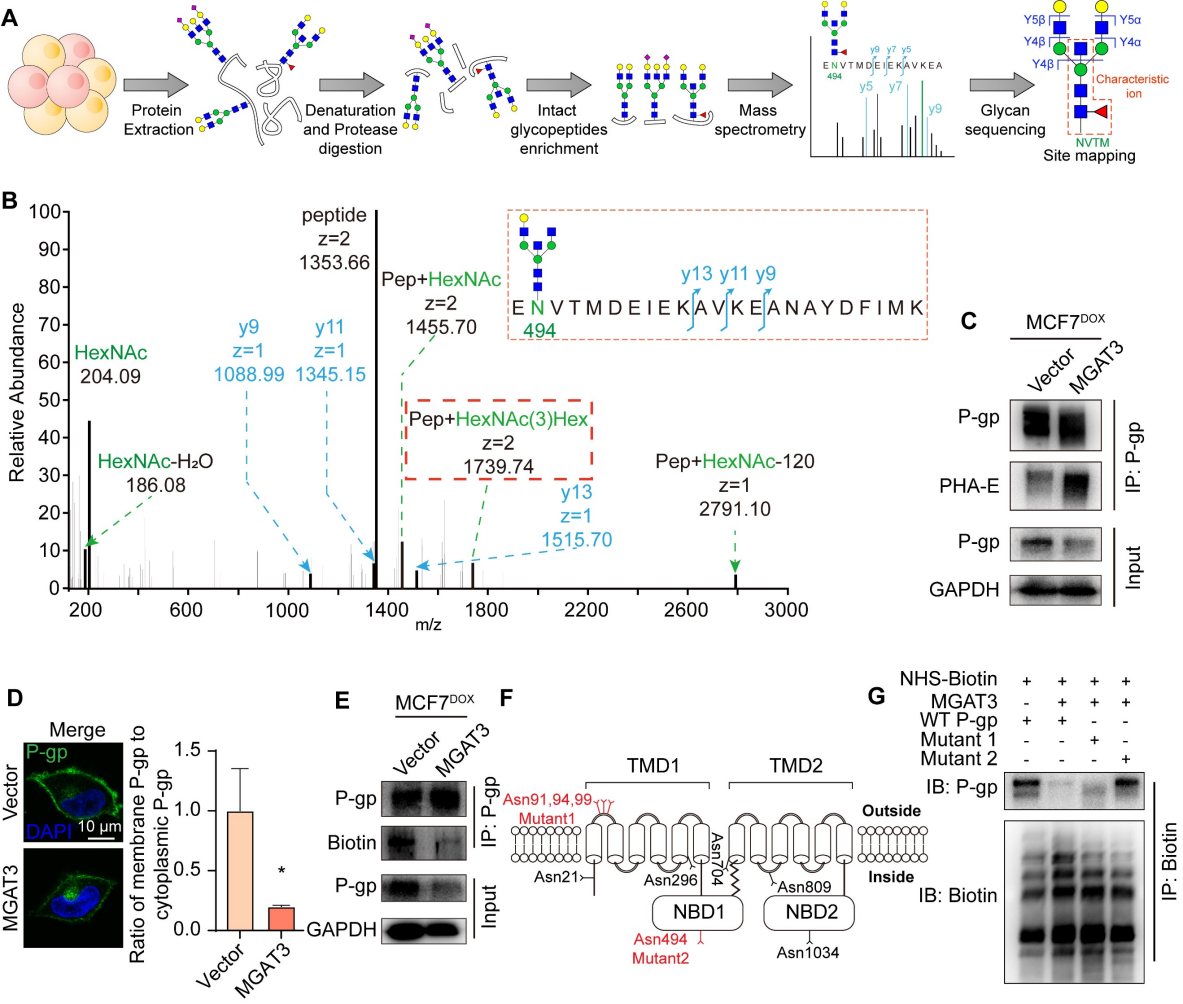
** Identification of P-gp as the target protein bearing bisecting GlcNAc.** (A) Intact glycoproteomic analysis of bisecting GlcNAc modified peptides by MAX enrichment and LC-MS/MS (schematic). (B) Representative MS/MS spectrum of P-gp peptide EN^#^VTMDEIEKAVKEANAYDFIMK with bisecting GlcNAc. #, glycosite. (C) Expression of P-gp with bisecting GlcNAc in parental and MGAT3 overexpressed MCF7^DOX^ cells determined by immunoprecipitation. (D) Localization of P-gp determined by immunofluorescence. (E) Membranous P-gp expression in parental and MGAT3 overexpressed MCF7^DOX^ cells labeled with NHS-LC-biotin. (F) Mutations of P-gp glycosites. (G) Membranous P-gp expression in NHS-LC-biotin labeled MDA-MB-231 cells transfected with P-gp, MGAT3 plus P-gp, MGAT3 plus P-gp Mut1, and MGAT3 plus P-gp Mut2 by immunoprecipitation. Membrane proteins were labeled with NHS-LC-biotin, as described in M&M, and subjected to immunoprecipitation with streptavidin-coated magnetic beads, followed western blotting with HRP-labeled streptavidin and antibody against P-gp.

With a combination of MAX enrichment and LC-MS/MS (**Fig. 4A**), the target glycoprotein, P-gp, was identified as bearing bisecting GlcNAc at Asn494 on intact glycopeptides by specific [pep+N3H] ions, among 47 bisecting GlcNAc attached glycopeptides (**Fig. 4B, S5A-C, Tab. S3**). P-gp was further validated to be modified with bisecting GlcNAc, and was down-regulated in MGAT3 overexpressed MCF7^DOX^ cells (**Fig. 4C**), without significant changes at the mRNA level (**Fig. S6A**).

The induction of P-gp expression by DOX treatment was attenuated by introduction of bisecting GlcNAc (**Fig. S6B**). With the incorporation of azidohomoalanine (AHA), an analog of methionine (**Fig. S6C**), the amount of newly synthesized P-gp was nearly equivalent in MCF7^DOX^ cells, regardless of bisecting GlcNAc levels (**Fig. S6D**). Furthermore, P-gp levels on the plasma membrane were reduced in MCF7^DOX^/MGAT3 cells (**Fig. 4D, S6E**). We further labeled membranous P-gp using NHS-LC-biotin (**Fig. S6F**), and found the presence of bisecting GlcNAc facilitated the degradation of membranous P-gp (**Fig. 4E, S6G**) through both proteasomal and lysosomal pathways (**Fig. S6H-J**), and inhibition of P-gp was correlated with reversal of chemoresistance (**Fig. S6K&L**). This suggests that the localization and degradation of P-gp but not its synthesis are primarily modulated by bisecting GlcNAc.

To investigate the effect of bisecting GlcNAc at given glycosites on the biological function of P-gp, mutant at conventional glycosites of Asn 91, 94 and 99 (termed as Mut1) and mutant at Asn 494 (termed as Mut2) were constructed **(Fig. 4F)**. In comparison with MB231/MGAT3 transfected with wild type P-gp or Mut1, glycosite deletion at Asn494 recovered membranous P-gp distribution on membrane (**Fig. 4G, S6M**), and retarded its degradation **(Fig. S6N&O)**, consequently leading to the reversion of cell apoptosis and viability **(Fig. S6P&Q)**. These findings indicate that the presence of site-specific bisecting GlcNAc may reverse chemotherapy resistance by regulating the localization and degradation of P-gp.

### The bisecting GlcNAc interferes the interaction of P-gp and ezrin

**Figure 5:**
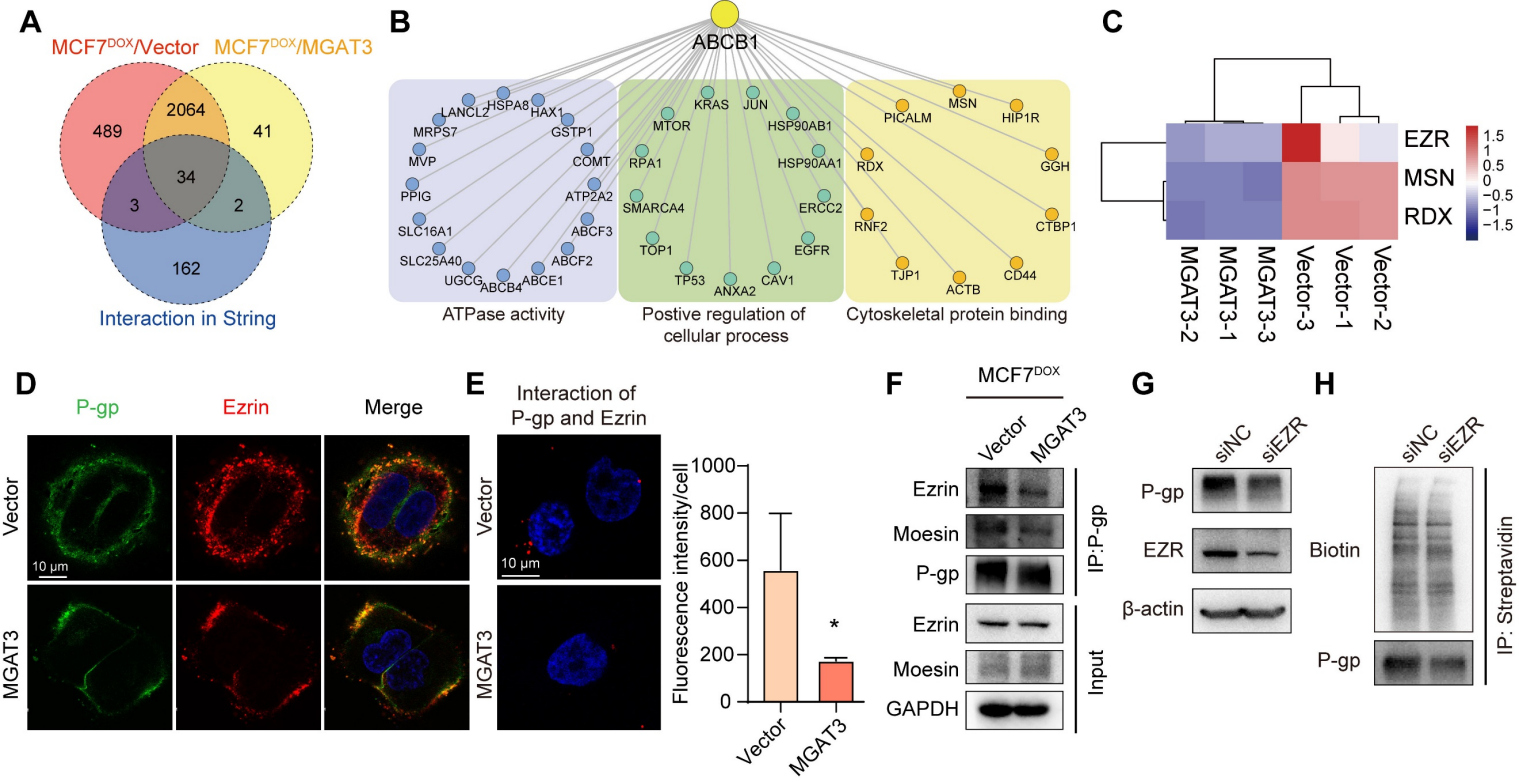
** P-gp-ezrin interaction reduced by bisecting GlcNAc.** (A) Venn diagram exhibiting P-gp interactome from STRING, and proteins differentially associated with P-gp in MCF7^DOX^/MGAT3 compared to MCF7^DOX^ cells. (B) Protein-protein interaction of 39 overlapped proteins. (C) Heatmap exhibiting ERM family interacted with P-gp. (D) Co-localization of P-gp and ezrin in parental and MGAT3 overexpressed MCF7^DOX^ cells. (E) Interaction of P-gp and ezrin detected by Duolink in situ proximity ligation assay. (F) The interaction between P-gp and ezrin/moesin determined by immunoprecipitation. (G) Total expression of P-gp and ezrin in ezrin silenced MCF7^DOX^ cells. (H) Membranous P-gp expression in ezrin silenced cells labeled with NHS-LC-biotin.

P-gp may interact with the actin cytoskeleton via ERM (ezrin/radixin/moesin) proteins, which serve as cross-linkers between cortical actin filaments and plasma membranes 32,33. Using IP-MS, we compared the proteins differentially associated with P-gp in MCF7^DOX^/MGAT3 compared to MCF7^DOX^ cells, and known P-gp interactome from STRING database (**Fig. 5A, S7A-C, Tab. S4**). These proteins were enriched for cytoskeletal protein binding, ATPase activity, and regulation of cellular processes (**Fig. 5B**). Of these, the interaction between P-gp and ERM proteins was suppressed by high bisecting GlcNAc levels (**Fig. 5C**). Specifically, P-gp was observed to be interacted with ezrin more than moesin (**Fig. 5D, S7D**), and the interaction was attenuated by the introduction of bisecting GlcNAc (**Fig. 5E&F**). Moreover, silencing ezrin reduced total and membranous P-gp expression (**Fig. 5G&H, S7E-G**), and promoted P-gp degradation (**Fig. S7H&I**), resulting in an increased apoptosis, followed by suppressed proliferation and cell viability in MCF7^DOX^ cells with DOX treatment (**Fig. S7J-L**). Moreover, in comparison with MB231/MGAT3 transfected with wild type P-gp or Mut1, glycosite deletion at Asn494 recovered the interaction between P-gp and ezrin (**Fig. S7M**). These data suggest that bisecting GlcNAc has the potential to affect the structural conformation of P-gp and disrupt its interaction with ERM proteins, ultimately resulting in a reduced expression of P-gp on the cell membrane.

### Bisecting GlcNAc attenuated chemoresistance transmission via a decreased P-gp in microvesicles

**Figure 6:**
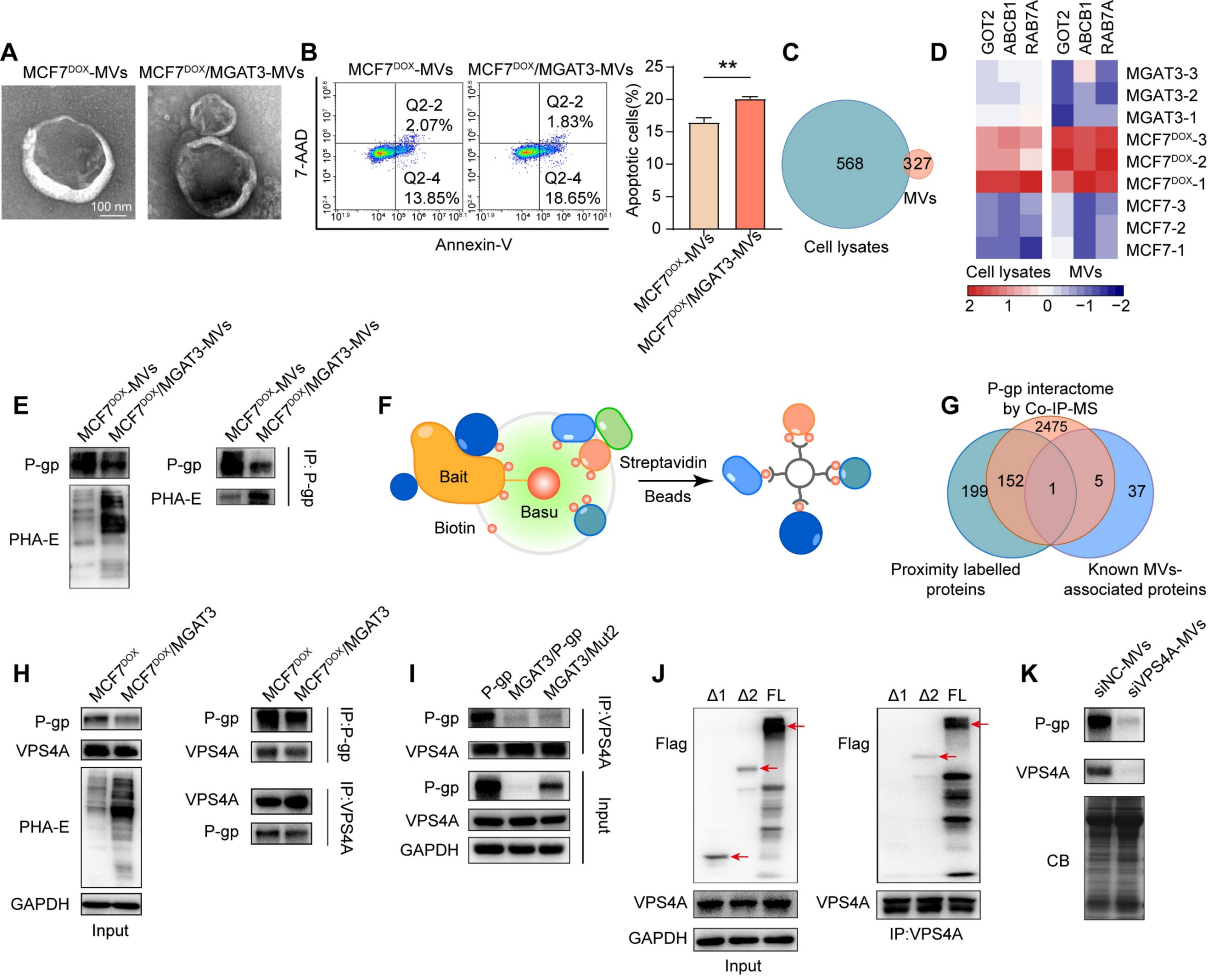
** Effects of bisecting GlcNAc on vesicular P-gp mediated chemoresistance transmission.** (A) Morphology of MCF7^DOX^-MVs and MCF7^DOX^/MGAT3-MVs. (B) Cell apoptosis of recipient cells treated with MCF7^DOX^-MVs and MCF7^DOX^/MGAT3-MVs. (C) Venn diagram of proteins showing a broadly increase in cell lysates and MVs of MCF7^DOX^ compared to MCF7, following by a decrease in which of MCF7^DOX^/MGAT3 compared to MCF7^DOX^. (D) Expression pattern of proteins showing overlapping expression patterns in cell lysates and MVs of MCF7, MCF7^DOX^ and MCF7^DOX^/MGAT3. (E) P-gp and its bisecting GlcNAc levels in MCF7^DOX^-MVs and MCF7^DOX^/MGAT3-MVs. (F) Proximity-dependent biotinylation of MV-associated proteins by fusion of Annexin A1 to Basu (schematic). (G) Venn diagram of proximity labeled proteins, P-gp interactome by Co-IP-MS, and known MV-associated proteins. (H) Interaction between P-gp and VPS4A in MCF7^DOX^ and MCF7^DOX^/MGAT3 cells. (I) Interaction between P-gp and VPS4A in cells transfected with P-gp, MGAT3 plus P-gp, and MGAT3 plus P-gp Mut2. (J) Interaction between VPS4A and full-length and truncated P-gp by immunoprecipitation. (K) P-gp levels in MVs from VPS4A silenced MCF7^DOX^ cells.

Based on the nature of microvesicles (MVs), accumulated studies demonstrated that MVs were responsible for the “non-genetic” acquisition of drug resistance 34. We found that drug resistance of recipient cells MDA-MB-231 was enhanced by soluble components from MCF7^DOX^ cells (**Fig. S8A&B**), but inhibited by which from MCF7^DOX^/MGAT3 cells (**Fig. S8C-E**). We isolated MVs from MCF7^DOX^ and MCF7^DOX^/MGAT3 cells (**Fig. 6A, S8F&G**), and found that bisecting GlcNAc clearly suppressed MCF7^DOX^-MV mediated chemoresistance transmission to recipient cells (**Fig. 6B**), but have no effect on secretion, morphology and uptake of MVs (**Fig. 6A, S8H&I**).

Proteomic analysis revealed that P-gp exhibited a significant increase in both cell lysates and MVs of MCF7^DOX^ compared to MCF7, followed by a decrease in which of MCF7^DOX^/MGAT3 compared MCF7^DOX^ (**Fig. 6C&D, S9A&B, Tab. S5&6**). Bisecting GlcNAc suppressed P-gp levels in MCF7^DOX^/MGAT3-MVs (**Fig. 6D&E**), consequently inhibiting the endocytosis and recycle of P-gp onto plasma membrane of recipient cells (**Fig. S9C**). The chemoresistance transmission could be eliminated by the P-gp inhibitor (**Fig. S9D**) or P-gp silencing (**Fig. S9E**).

To explore the protein responsible for the vesicular P-gp recruitment, a proximity-dependent labelling was performed using Annexin A1, a specific marker of MVs 35, as the bait (**Fig. 6F, S10A&B, Tab. S7**). VPS4A was identified as a candidate by overlapping proximity labeled proteins, P-gp interactome by Co-IP-MS (**Tab. S4**), and known MV associated proteins 36-38 (**Fig. 6G**). The interaction between P-gp and VPS4A was suppressed by introduction of bisecting GlcNAc using Co-IP (**Tab. S4**) and immunoprecipitation (**Fig. 6H**), and which was recovered by deletion of site-specific bisecting GlcNAc of P-gp (**Fig. 6I**). Notably, VPS4A was associated with the full length and truncated P-gp Δ1 but not with the truncated P-gp Δ2 (**Fig. 6J, S10C**), indicating that the NBD1 domain which contains bisecting GlcNAc site (Asn494), was responsible for their interaction. Silencing VPS4A in donor cells clearly reduced levels of vesicular P-gp (**Fig. 6K, S10D**), and drug resistance in recipient cells treated with soluble compounds (**Fig. S10E**), indicating a regulatory role of VPS4A in vesicular P-gp recruitment.

### Structural transformation of P-gp induced by bisecting GlcNAc

**Figure 7:**
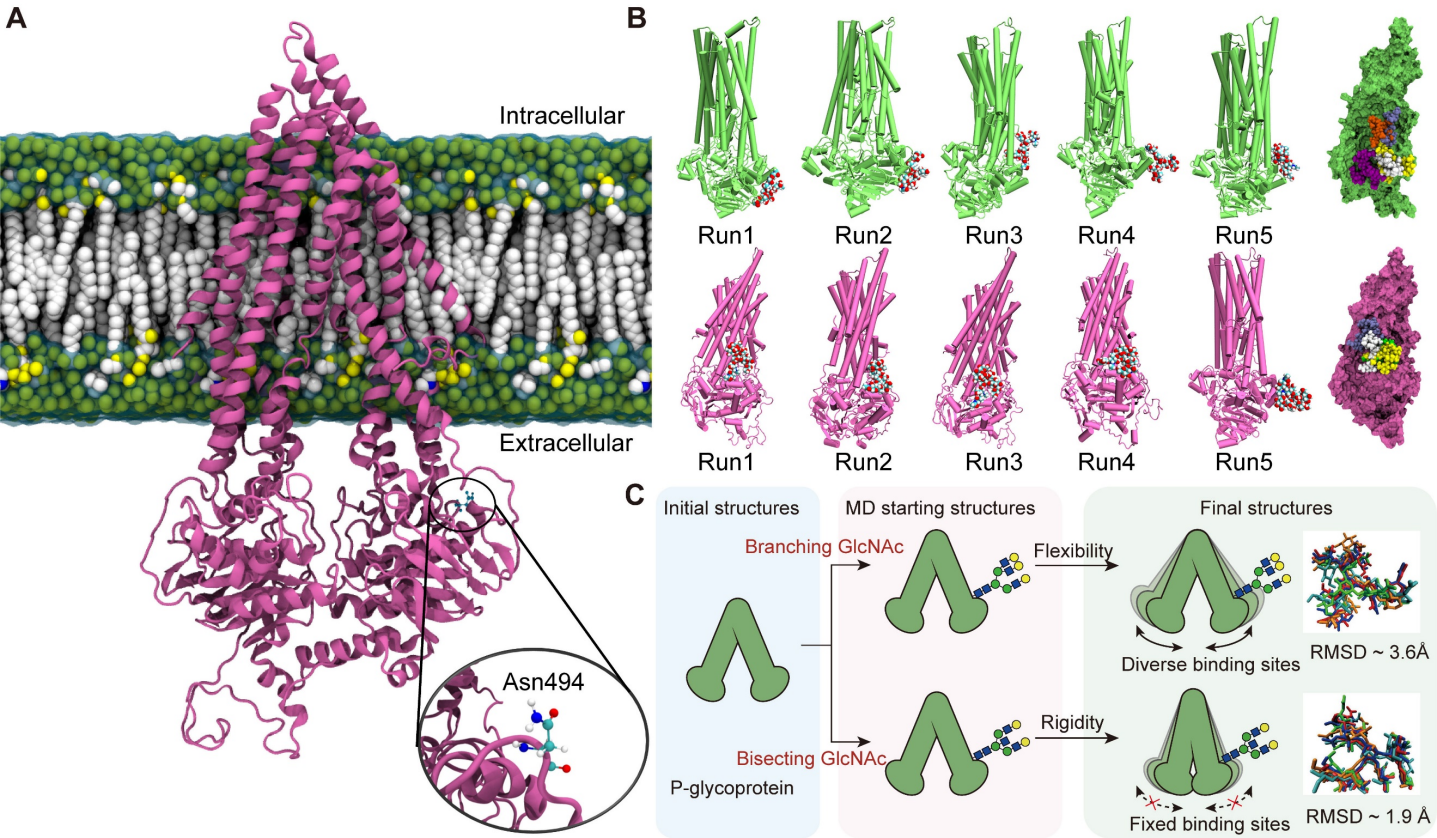
** Effects of bisecting GlcNAc on global and local structure of P-gp.** (A) The initial structure of P-gp embedded in cell membrane is shown, with Asn494 highlighted. (B) The final frames of branching GlcNAc (lime) and bisecting GlcNAc (mauve) decorated P-gp are depicted. Branching and bisecting GlcNAc are shown as van der Waals balls, with hydrogen, oxygen, carbon and nitrogen atoms in white, red, cyan and blue, respectively. The relative positions of GlcNAc are indicated by the superpositions of P-gp with branching GlcNAc or bisecting GlcNAc shown in different colors. (C) The structural basis for the suppression of P-gp activity induced by bisecting GlcNAc.

As the expression of MGAT3 enhances, the amount of bisecting GlcNAc increases while the amount of β1,6 GlcNAc branching GlcNAc decreases 39. To model the effect of these changes, atomic coordinates of P-glycoprotein with branching or bisecting GlcNAc structures attached to the nitrogen atom of Asn494 were generated (**Fig. 7A, S11A&B**). The RMSFs patterns observed in analogous regions in the three types of P-gp (non-glycosylated, branching, and bisecting GlcNAc glycosylated) were similar, highlighting the conservation of structural dynamics in P-gp (**Fig. S12&13**).

Although N-glycosylation at Asn494 does not significantly affect the global and local structure of P-gp, it is noteworthy that the proximity of bisecting GlcNAc and branching GlcNAc to the P-gp protein interface showed significant changes (**Fig. 7B**). Compared to branching GlcNAc, the bisecting GlcNAc remained in similar regions throughout four of the five simulations, which "saddle stitch" the hinge-like staples (**Fig. 7C**). Hydrogen bonding interactions are the main driving force behind the binding of GlcNAc and P-gp, which involves polar residues distributed on the surface of P-gp (**Fig. S14A&B**). The average occupancy of hydrogen bonds has also been calculated, and the distribution of hydrogen bonds formed between branching GlcNAc and P-gp is more dispersed compared to that of bisecting GlcNAc (**Fig. S14C&D**), which further validates the diversity of the branching GlcNAc binding patch.

## Discussion

It is known that glycans act essential roles in cancer development, progression, and chemoresistance. Our findings here demonstrated that bisecting GlcNAc levels were suppressed in chemoresistant BC cells, and forced enhancement of bisecting GlcNAc by introducing MGAT3 partially reverse chemoresistance, consistent with the anti-chemoresistance role MGAT3 in lymphoblastoid cell line 40. Using mass spectrometric analysis of glycoproteins, we identified P-gp as a target protein bearing bisecting GlcNAc in breast cancer cells.

As one typical glycoprotein, P-gp extrudes drugs from living cells, thereby conferring resistance to chemotherapy. Inhibition of N-glycosylation with tunicamycin facilitated daunorubicin accumulation and reversed chemoresistance by reduced surface-associated P-gp in colorectal cancer cells 41. Similarly, silencing of ribopholin II (RPN2), which is a subunit of central enzyme of N-glycan oligosaccharyltransferase (OST), could decrease the N-glycosylation on P-gp to inhibit its expression on the cell surface, resulting in sensitizing to drugs 42. These results aligned with the fact that only fully N-glycosylated isoforms of the transporters (including P-gp, ABCC2 and ABCG2) were associated with functional activity, and the absence of the glycan trapped the transporters in subcellular compartments 43. P-gp was found to be decorated with high mannose type N-glycans recognized by *Galanthus nivalis* agglutinin (GNA), α2,3 and α2,6-linked sialic acids recognized by *Sambucus nigra* (SNA) and *Maackia amurensis* (MAA) lectins, and branched GlcNAc recognized by *Datura stramonium* (DSA) in uterine sarcoma human cell line MES-SA/D × 5 44. Among these terminal modification, sialic acids and branched GlcNAc were documented to be suppressed by bisecting GlcNAc 45, suggesting that bisecting GlcNAc attenuated the membrane localization of P-gp via the inhibition of terminal modification on P-gp. Consistently, our current study revealed that bisecting GlcNAc modification did not affect the newly synthesized P-gp, but instead decreased its expression and membrane localization, leading to a weakened ability to resist chemotherapy. These findings provide further evidence for the significant impact of glycosylation on P-gp degradation and localization, as previously demonstrated 40.

While there are ten potential consensus sequences for N-glycosylation, only three specific N-glycosites (Asn91, 94, and 99) located in the first extracellular loop have been reported to undergo glycosylation 46. Mutants of these conventional glycosites (N91, 94, 99Q) exhibited reduced expression of P-gp on the cell membrane, but the half-life of the mutated P-gp remained unchanged 47. In other study, Asn99 mutated P-gp exhibited markedly reduced the efflux pump function in gastric cancer cells 10. Interestingly, our study identified an additional bisecting GlcNAcylation site at Asn494 in the intracellular domain of P-gp (**Fig. 4B**). Although N-GlycositeAtlas database documents the presence of this glycosite at Asn494 48, its glycosylation status and function have not yet been investigated. We found that the removal of bisecting GlcNAc at Asn494, rather than the conventional glycosites Asn91, 94 and 99, restored P-gp expression and chemoresistance in MGAT3 overexpressed MCF7^DOX^ cells, indicating the pivotal role of bisecting GlcNAc at Asn494 in regulating P-gp expression and its ability to confer chemoresistance.

Growing evidence has demonstrated that the localization and drug efflux function of P-gp are determined by its interactions with actin through ezrin/radixin/moesin (ERM) proteins 32,49. In our study, ezrin was identified to be interacted with P-gp in chemoresistant BC cells using Co-IP-MS. The expression of membranous P-gp and association between P-gp and ezrin were reduced in MCF7^DOX^ cells with high bisecting GlcNAc levels**,** *vice versa,* silencing ezrin impaired the location of P-gp onto membrane and attenuated the chemoresistance. Based on these observations, we propose that bisecting GlcNAc modification may affect the P-gp conformation on the cell membrane.

Except for the modulation of P-gp expression at either pre- or post-transcriptional levels, a “non-genetic” basis for the acquisition and spread of P-gp via extracellular vesicle transfer has been reported 34,50. MVs (10-1000 nm), produced by budding and blebbing from the plasma membrane, are abundant in cytosolic and membrane proteins. Importantly, the cargo within MVs is not randomly distributed; instead, selective recruitment of specific protein cargo into MVs is regulated by ARF6/VAMP3 51,52. In our study, we identified VPS4A as a critical factor responsible for recruiting P-gp into MVs, as evidenced by the reduction of vesicular P-gp levels upon VPS4A silencing. Furthermore, recent studies have revealed that proteins destined for sorting into MVs must first be targeted to the plasma membrane 37, and VPS4A has been implicated in regulating plasma membrane localization 53, indicating a potential mechanism of VPS4A-mediated MVs cargo recruitment. Notably, our findings demonstrated that bisecting GlcNAc hinders the interaction between P-gp and VPS4A, leading to a decrease in vesicular P-gp levels and subsequently inhibiting MVs-mediated chemoresistance transmission. This suggests that specific N-glycans, such as bisecting GlcNAc, act as determinants for sorting vesicular cargo, offering new insights into the regulation of MVs cargo composition and its impact on chemoresistance.

The continuously developing techniques including X-ray crystallography, nuclear magnetic resonance (NMR) and Cryo-electron microscopy (cryo-EM), have enabled the resolution of the structures of numerous proteins 54,55. Nevertheless, the X-ray crystallography structures of these proteins that have been resolved usually lack glycans, as these are not discernible due to their high flexibility and microheterogeneity. Therefore, it is still impractical to achieve in situ visualization of protein glycosylation, and decoding glycan heterogeneity is challenging for understanding glycoprotein structure and function 56. The structure of P-gp was first resolved in 2009 by X-ray crystallography 57, revealing that the two NBDs formed a large internal cavity that can accommodate at least two substrates simultaneously 57. A more refined crystal structure of P-gp was obtained using de novo model building a selected region, exhibiting a more refined protein geometry 58. In this study, we docked bisecting GlcNAc and branching GlcNAc structures to the Asn494 site on P-gp by utilizing structural simulations.

The diversity of branching GlcNAc's binding sites is originated from its flexibility. In contrast with bisecting GlcNAc, the conformational space of branching GlcNAc is larger due to its asymmetry, which indicate that it has the propensity to accommodate more possible binding sites. This hypothesis has been further verified by the RMSD values of the two glycans. The ensemble averaged RMSD of branching GlcNAc is about 3.6 Å while the RMSD of bisecting GlcNAc is about 1.9 Å, which validate the structural diversity of branching GlcNAc. The regular binding sites of bisecting GlcNAc may contribute significantly to suppressing of P-gp activity. It has been reported that the dynamic nature of P-gp is indispensable to facilitate the unidirectional transport of substrates and local conformational flexibility around the lateral gate is essential to assist substrate entry from the inner leaflet of the membrane 59. We proposed that the presence of bisecting GlcNAc can introduce structural constraints that force the P-gp into a state not suitable for further allostery, which may suppress the activity of P-gp. In contrast with bisecting GlcNAc, the more flexible branching GlcNAc is less likely to interact with the similar binding sites, which has little effect on the functionality of P-gp. These data suggest that the role of glycans on target protein is specific, and changes in their structures may fine-tune protein function.

## Conclusion

In conclusion, our study demonstrate that bisecting GlcNAc modification reversed the chemoresistance by unflexing the conformation of P-gp, accelerating its degradation, and suppressing its recruitment into MVs. While our study integrated protein glycosylation models with multi-omics approaches and a structural dynamic strategy, the intricate link between P-gp function and specific glycan structures still requires emerging methods for further exploration.

## Supplementary Material

Supplementary figures and table legends.

Supplementary table 1.

Supplementary table 2.

Supplementary table 3.

Supplementary table 4.

Supplementary table 5.

Supplementary table 6.

Supplementary table 7.
