# Supplementary material for: Bisecting GlcNAc modification reverses the chemoresistance via attenuating the function of P-gp
Source: Theranostics. 2024 Aug 19;14(13):5184–99. doi: 10.7150/thno.93879 (PMC11388069; doi:10.7150/thno.93879)
Supplement: Supplementary file 1 — Supplementary figures and table legends. [file thnov14p5184s1.pdf]

Supporting Information

Bisecting GlcNAc modification reverses the chemoresistance via attenuating the function of P-gp

Zengqi Tan[1,#], Lulu Ning[2,#], Lin Cao[1,#], Yue Zhou[3], Jing Li[3], Yunyun Yang[3], Shuai Lin[4], Xueting Ren[4], Xiaobo Xue[3], Huafeng Kang[4], Xiang Li[1,*], Feng Guan[3,*]

1 Institute of Hematology, Provincial Key Laboratory of Biotechnology, School of Medicine, Northwest University, Xi'an, Shaanxi, 710069, P.R. China

2 College of Bioresources Chemical and Materials Engineering, Shaanxi University of Science & Technology, Xi'an, Shaanxi, 710069, P.R. China

3 Key Laboratory of Resource Biology and Biotechnology in Western China, Ministry of Education, Provincial Key Laboratory of Biotechnology, College of Life Sciences, Northwest University, Xi'an, Shaanxi, 710069, P.R. China

4 Department of Oncology, The Second Affiliated Hospital of Xi'an Jiaotong University, Xi'an, Shaanxi, 710069, China.

[#] These three authors contributed equally

* Corresponding authors

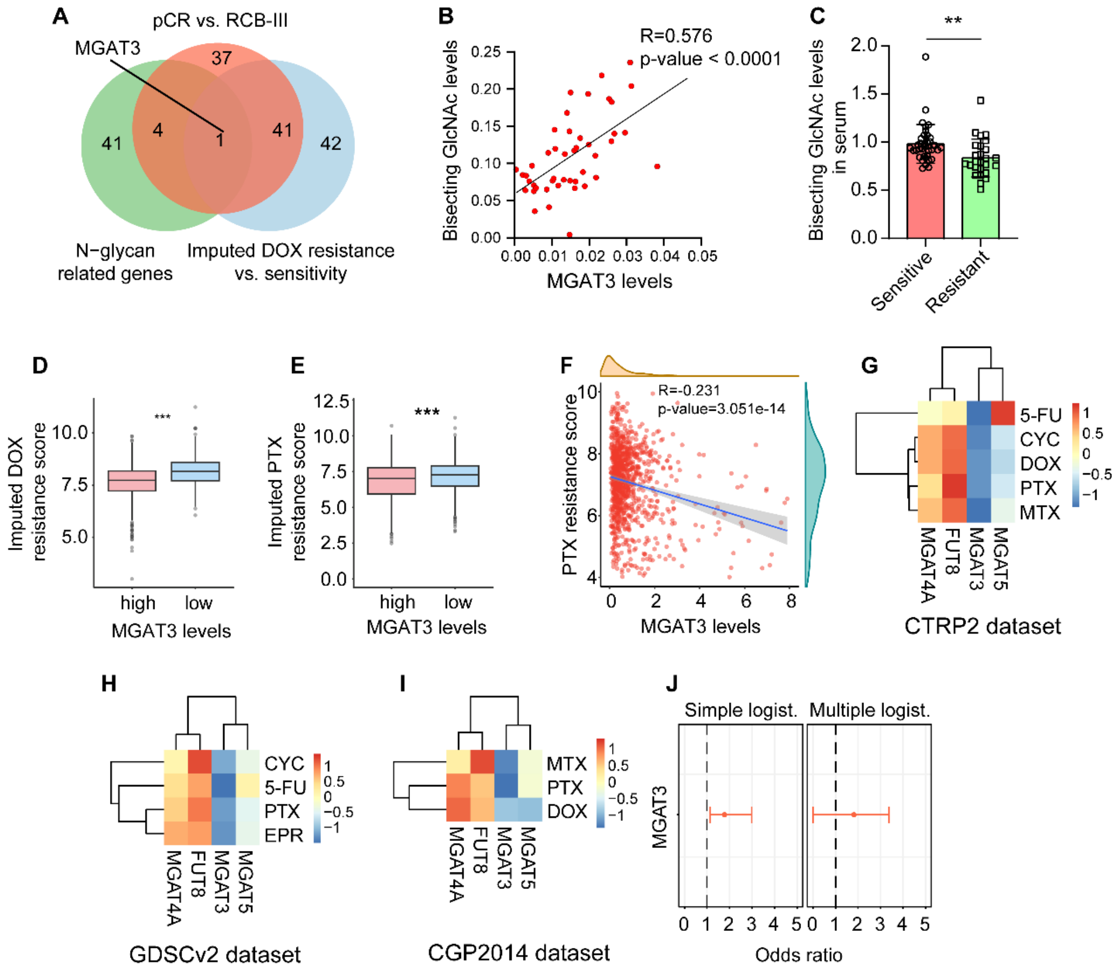

Figure S1. The correlation between MGAT3 and chemoresistance in BC

(A) Venn diagram of differentially expressed glycan-related genes in RCB-III groups compared to pCR groups, and which in imputed DOX resistant groups compared to sensitive groups from TCGA datasets by oncoPredict, and N-glycan-related genes from KEGG database. (B) Correlation between MGAT3 expression and bisecting GlcNAc levels in BC tissues post- and pre-NACT. (C) Bisecting GlcNAc levels in serum from chemosensitive and -resistance BC patients. (D&E) Imputed DOX (D) and PTX (E) resistance score in high- and low-MGAT3 expressed BC patients from TCGA datasets using oncoPredict package. High imputed drug resistance score indicates high drug resistance. (F) Correlation analysis of MGAT3 mRNA expression and imputed PTX resistance score in BC patients from TCGA database. (G-I) Correlation between tumor-associated glycosyltransferases expression and imputed drug resistance score across multiple training datasets: The Cancer Therapeutics Response Portal (CTRP), Genomics of Drug Sensitivity in Cancer (GDSC) and Cancer Genome Project (CGP). (J) Associations of MGAT3 mRNA with pCR using both simple and multiple logistic regression. Significant associations (P <

0.05, logistic regression) are shown in red. The parameter estimate is used as the measure of center, with error bars representing 95% confidence intervals.

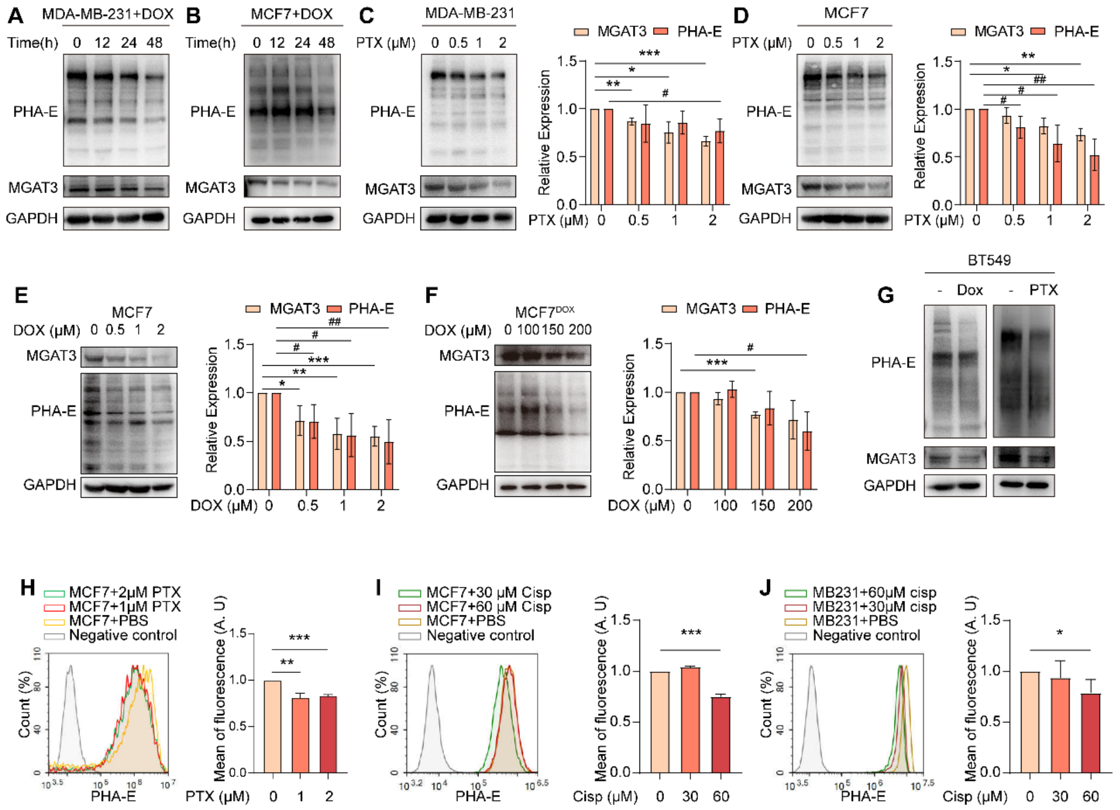

Figure S2. Bisecting GlcNAc levels and MGAT3 expression in BC cells upon DOX or PTX treatment

(A-F) Bisecting GlcNAc levels and MGAT3 expression in BC cells, including MDA-MB-231, MCF7 and MCF7^{DOX}, with DOX or PTX treatment for 48 h, determined by western and lectin blotting. (G) Bisecting GlcNAc levels and MGAT3 expression in BT549 cells treated with 2 μM DOX or 2 μM PTX for 48 h. (H-J) Bisecting GlcNAc levels in MDA-MB-231 and MCF7 cells with PTX or cisplatin (Cisp) treatment for 48 h, determined by flow cytometry.

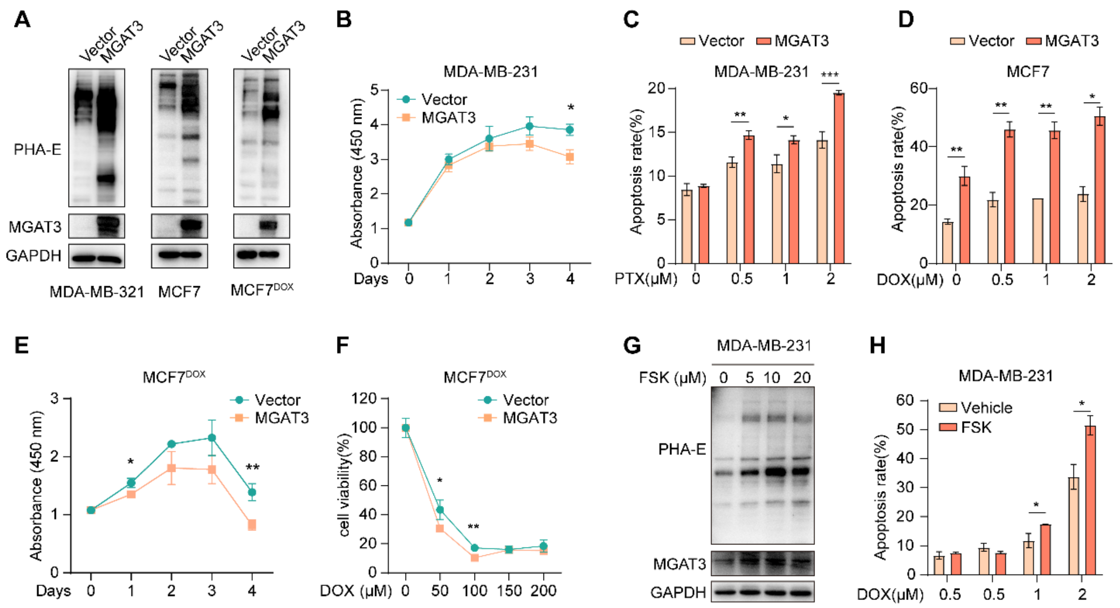

Figure S3. Bisecting GlcNAc levels and MGAT3 expression in BC cells upon forskolin treatment

(A) Bisecting GlcNAc levels and MGAT3 expression in MGAT3 overexpressed MDA-MB-231, MCF7 and MCF7^{DOX} cells. (B) Cell proliferation of parental and MGAT3 overexpressed MDA-MB-231 cells with DOX treatment. (C) Cell apoptosis of parental and MGAT3 overexpressed MDA-MB-231 cells with PTX treatment. (D) Cell apoptosis of parental and MGAT3 overexpressed MCF7 cells with DOX treatment. (E&F) Cell proliferation (E) and viability (F) assays of parental and MGAT3 overexpressed MCF7^{DOX} cells with DOX treatment. (G) Bisecting GlcNAc levels and MGAT3 expression in MDA-MB-231 cells with FSK treatment. (H) Cell apoptosis of DOX-exposed MDA-MB-231 cells with/ without FSK treatment.

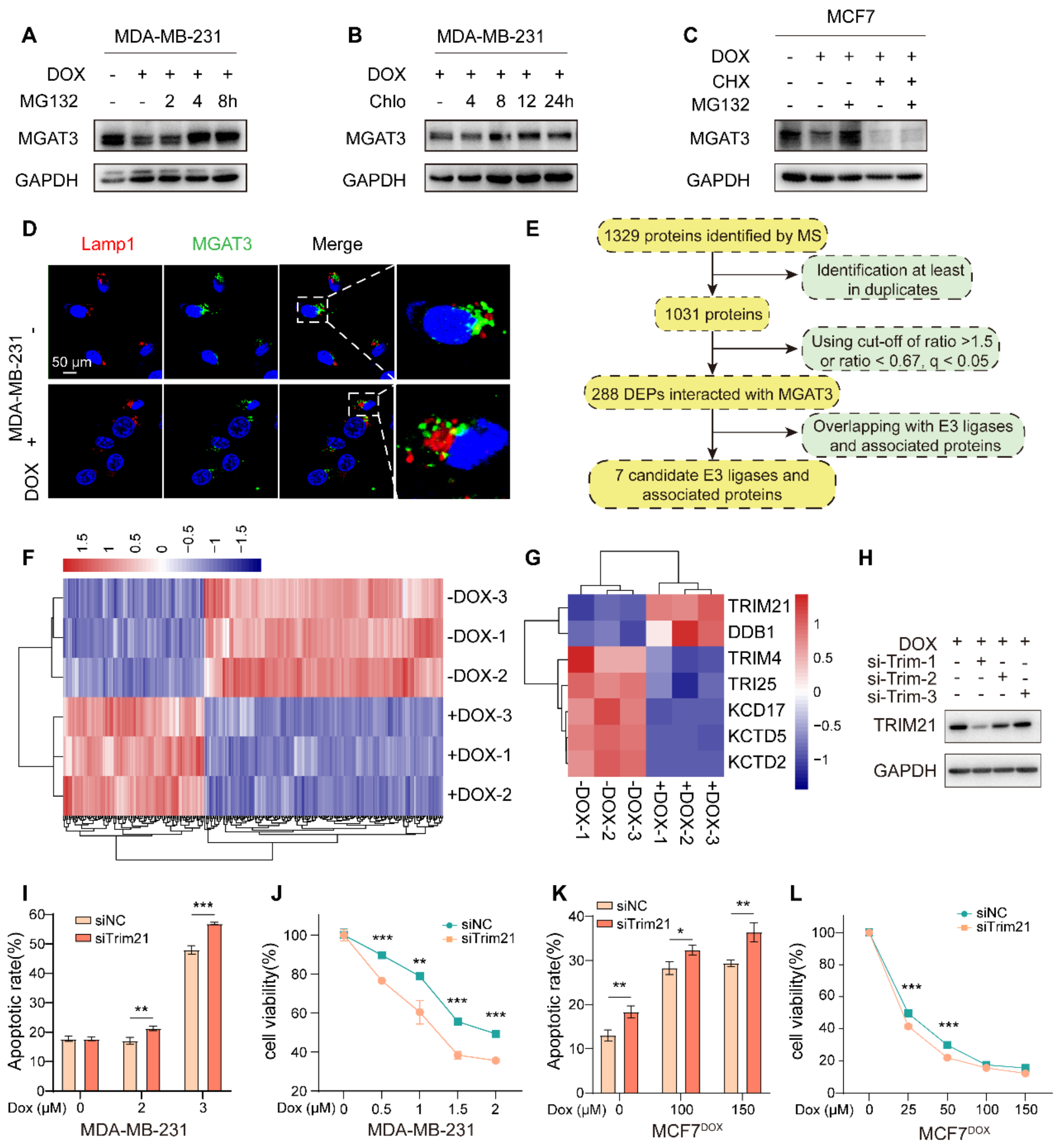

Figure S4. MGAT3 degradation in BC cells upon chemotherapy drug treatment

(A&B) MGAT3 expression in MDA-MB-231 cells treated with DOX in the presence of MG132 (A) or Chlo (B). (C) MGAT3 expression in MCF7 cells treated with DOX in the presence of MG132 or Chlo. (D) Localization of MGAT3 in cells treated with/ without DOX. Red: lamp1, the lysosome marker. Green: MGAT3. Blue: nucleus. Scale bar: 50 mm. (E) Data analysis of Co-IP-MS with antibody against MGAT3. (F) Heatmap exhibiting proteins differentially interacted with MGAT3 in MDA-MB-231 treated with/ without DOX. (G) Expression pattern of E3 ligases and related proteins differentially interacted with MGAT3. (H) Trim21 silencing in MDA-MB-231 cells. (I-L) Cell apoptosis and viability assays of Trim21 silenced MDA-MB-231 (I&J) and MCF7^{DOX} cells (K&L).

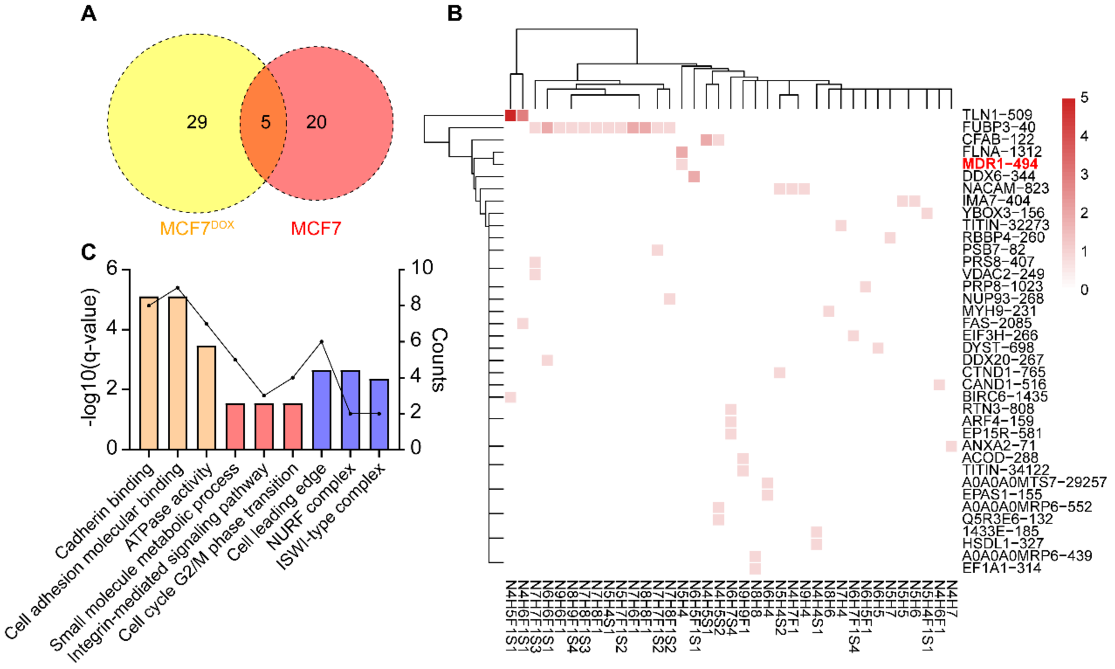

Figure S5. Glycopeptides with bisecting GlcNAc identified in MCF7 and MCF7^{DOX} cells

(A) Venn diagram exhibiting glycopeptides with bisecting GlcNAc identified in MCF7 and MCF7^{DOX} cells. (B) Heatmap exhibiting identified bisecting N-glycans on intact glycopeptides with PSMs number. Peptide-spectrum-matches of the intact glycopeptides, comprising of different glycans (bottom) and their glycosite locations in different glycoproteins (right) are exhibited. (C) Enriched GO analysis of identified glycoproteins with bisecting GlcNAc.

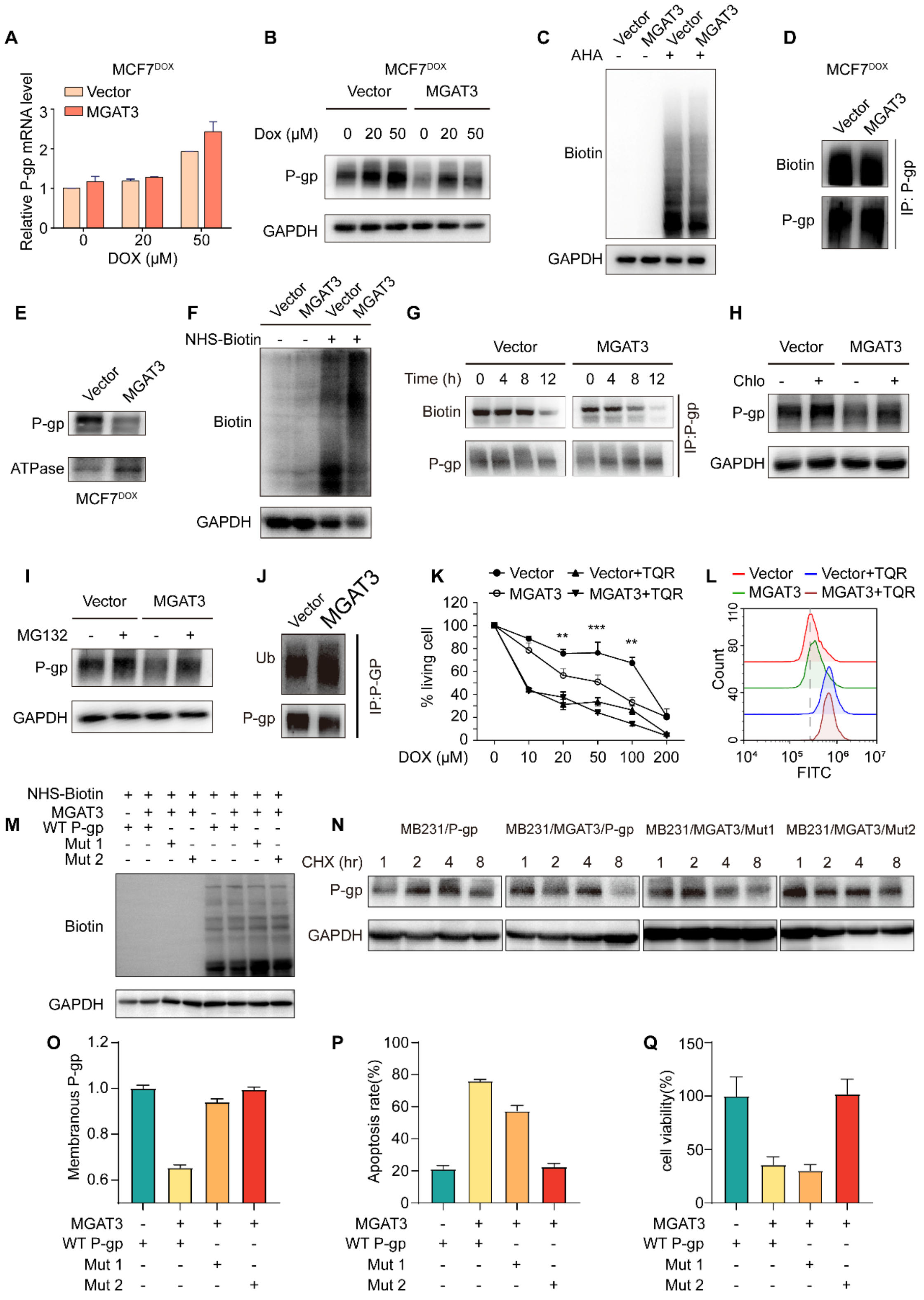

Figure S6. Effects of bisecting GlcNAc on P-gp biological function and expression

(A) P-gp mRNA expression relative to β-actin in parental and MGAT3 overexpressed MCF7^{DOX} cells. (B) P-gp expression in parental and MGAT3

overexpressed MCF7^{DOX} cells treated with different doses of DOX for 48 h. (C) The expression of newly synthesized proteins labeled with biotin. (D) The expression of newly synthesized P-gp by immunoprecipitation. (E) P-gp expression in membrane fraction. (F) The expression of membranous proteins labeled with NHS-LC-biotin. (G) Half-life of membranous P-gp labeled with NHS-LC-biotin in parental and MGAT3 overexpressed MCF7^{DOX} cells exposed to CHX by immunoprecipitation with streptavidin-coated magnetic breads and western blotting with HRP-streptavidin. (H&I) P-gp expression in parental and MGAT3 overexpressed MCF7^{DOX} cells treated with Chlo (H) or MG132 (I). (J) Polyubiquitination levels on P-gp by immunoprecipitation and western blotting. (K&L) Cell viability (K) and drug efflux (L) assays of parental and MGAT3 overexpressed MCF7^{DOX} cells treated with/ without P-gp inhibitor, TQR. (M) The expression of membranous proteins labeled with NHS-LC-biotin in MDA-MB-231 cells transfected with P-gp, MGAT3 plus P-gp, MGAT3 plus P-gp Mut1, and MGAT3 plus P-gp Mut2. Membrane proteins were labeled with NHS-LC-biotin, as described in M&M, and subjected to western blotting with HRP-labeled streptavidin. (N) Half-life of membrane P-gp labeled with NHS-LC-biotin in constructed mutants exposed to CHX. (O) The expression of membranous P-gp determined by flow cytometry analysis. (P&Q) Cell apoptosis (P) and viability (Q) assays of constructed mutants.

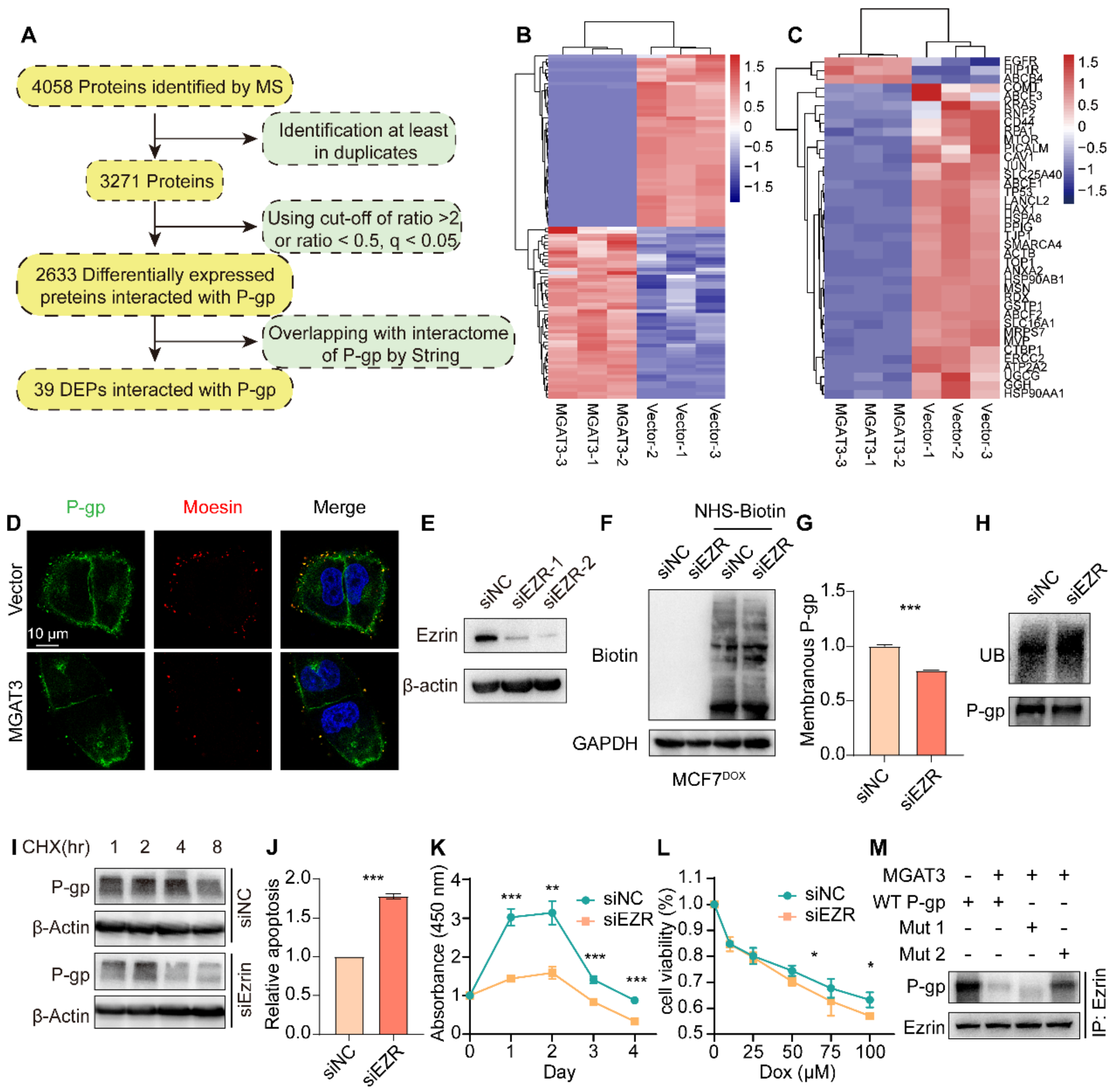

Figure S7. Identification of the interaction between ezrin and P-gp

(A) Data analysis process of Co-IP-MS with antibody against P-gp. (B) Heatmap exhibiting identified proteins differentially interacted with P-gp in MGAT3 overexpressed MCF7^{DOX} cells compared to parental cells. (C) Heatmap exhibiting identified proteins which were both differentially interacted with P-gp, and annotated as P-gp interactome from STRING. (D) Co-localization of P-gp and moesin in parental and MGAT3 overexpressed MCF7^{DOX} cells. (E) Ezrin silencing in MCF7^{DOX} cells. (F) The expression of membranous proteins labeled with NHS-LC-biotin. (G) The expression of membranous P-gp determined by flow cytometry. (H) Polyubiquitination levels on P-gp in ezrin silenced MCF7^{DOX} cells. (I) Half-time of P-gp in CHX-exposed parental and ezrin silenced MCF7^{DOX} cells. (J-L) Cell apoptosis (J), proliferation (K) and viability (L) assays of parental and ezrin silenced MCF7^{DOX} cells. (M) The interaction between P-gp and ezrin in constructed mutants.

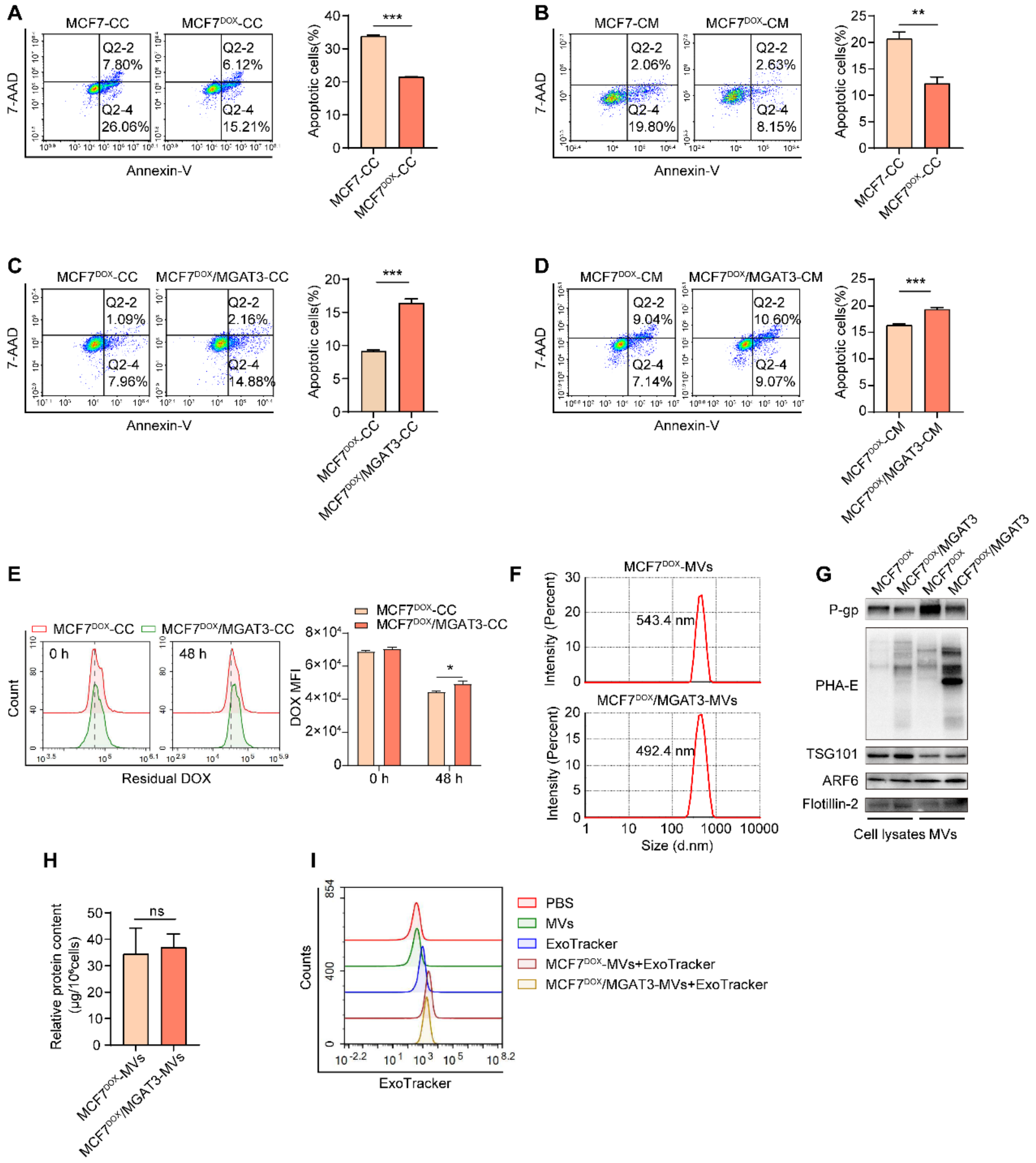

Figure S8. Effects of bisecting GlcNAc on soluble components mediated chemoresistance transmission

(A&B) Cell apoptosis of recipient cells MDA-MB-231 treated with soluble components from MCF7 and MCF7^{DOX} cells using co-culture (CC) system (A) and conditioned medium (CM) treatment (B). (C&D) Cell apoptosis of recipient cells treated with soluble components from MCF7^{DOX} and MCF7^{DOX}/MGAT3 cells using CC system (C) and CM treatment (D). (E) Drug efflux assay of recipient cells co-cultured with MCF7^{DOX} and MCF7^{DOX}/MGAT3 cells. (F-I) Particle diameter (F), marker expression (G), secretion (H), and uptake (I) of MCF7^{DOX-}MVs and MCF7^{DOX}/MGAT3-MVs.

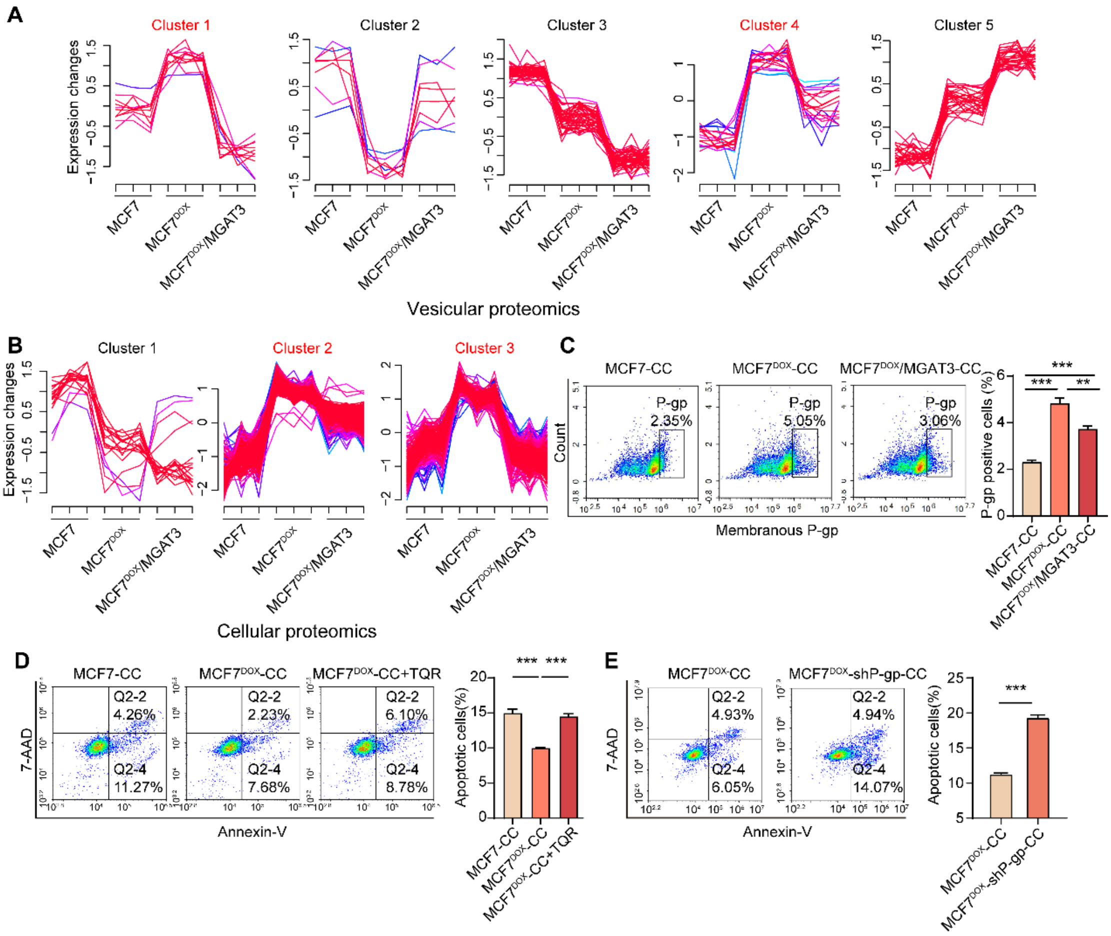

<u>Figure S9 Effects of bisecting GlcNAc on vesicular P-gp-mediated chemoresistance transmission</u>

(A&B) Mfuzz analysis of differentially expressed proteins from cell lysates (A) and MVs (B) in two comparison: MCF7^{DOX} vs. MCF7 and MCF7^{DOX}/MGAT3 vs. MCF7^{DOX}. (C) Membranous P-gp levels of recipient cells co-cultured with MCF7, MCF7^{DOX} and MCF7^{DOX}/MGAT3 cells. (D) Cell apoptosis of recipient cells co-cultured with MCF7^{DOX} cells with P-gp inhibitor TQR. (E) Cell apoptosis of recipient cells co-cultured with P-gp silenced MCF7^{DOX} cells.

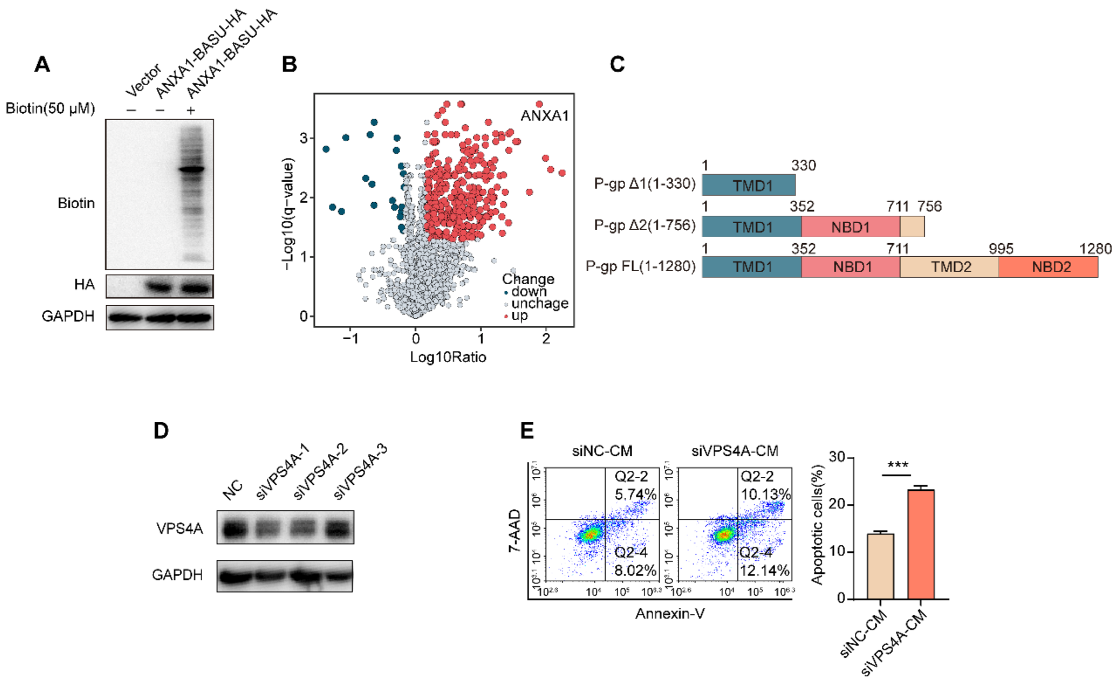

Figure S10. Regulatory role of VPS4A in MVs sorting

(A) Proximity biotinylation and HA levels in Annexin A1-Basu-HA transfected HEK293T cells incubated with/ without biotin. (B) Expression pattern of proximity labeled proteins differentially expressed in Annexin A1-Basu-HA transfected HEK293T cells incubated with/ without biotin. (C) Schematic description of full length (FL) and truncated mutants P-gp including Δ1 and Δ2. (D) VPS4A expression in VPS4A silenced MCF7^{DOX} cells. (E) Cell apoptosis of recipient cells treated with CM from VPS4A silenced cells.

A

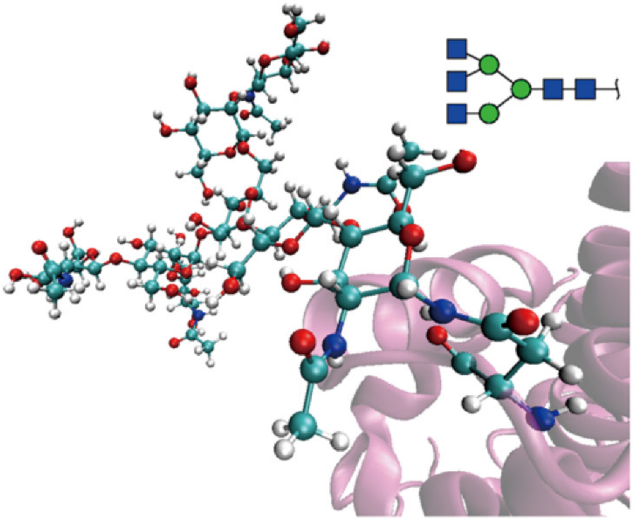

DGlcpNAcb1-2DManpa1-6[DGlcpNAcb1-4[DGlcpNAcb1-2]DManpa1-3]DManpb1-4DGlcpNAcb1-4DGlcpNAca1-OASN

B

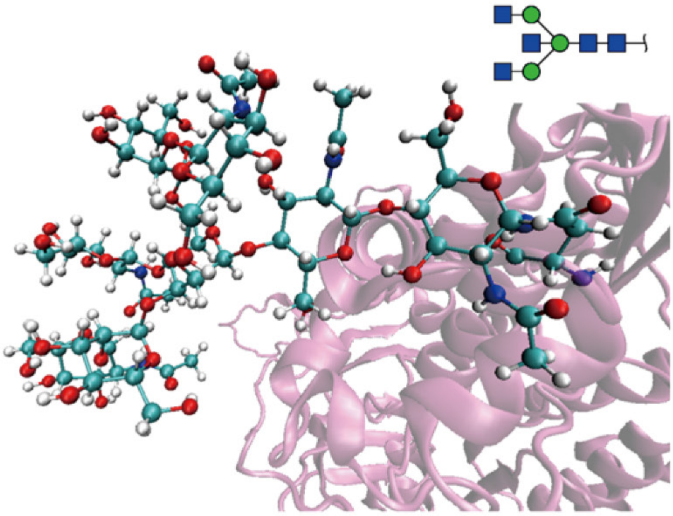

DGlcpNAcb1-2DManpa1-6[DGlcpNAcb1-4][DGlcpNAcb1-2DManpa1-3]DManpb1-4DGlcpNAcb1-4DGlcpNAca1-OASN

Figure S11. Structures of branching GlcNAc (A) and bisecting GlcNAc (B) displaying their three-dimensional conformations, sequences, and Symbol Nomenclature for Glycans (SNFG) representation

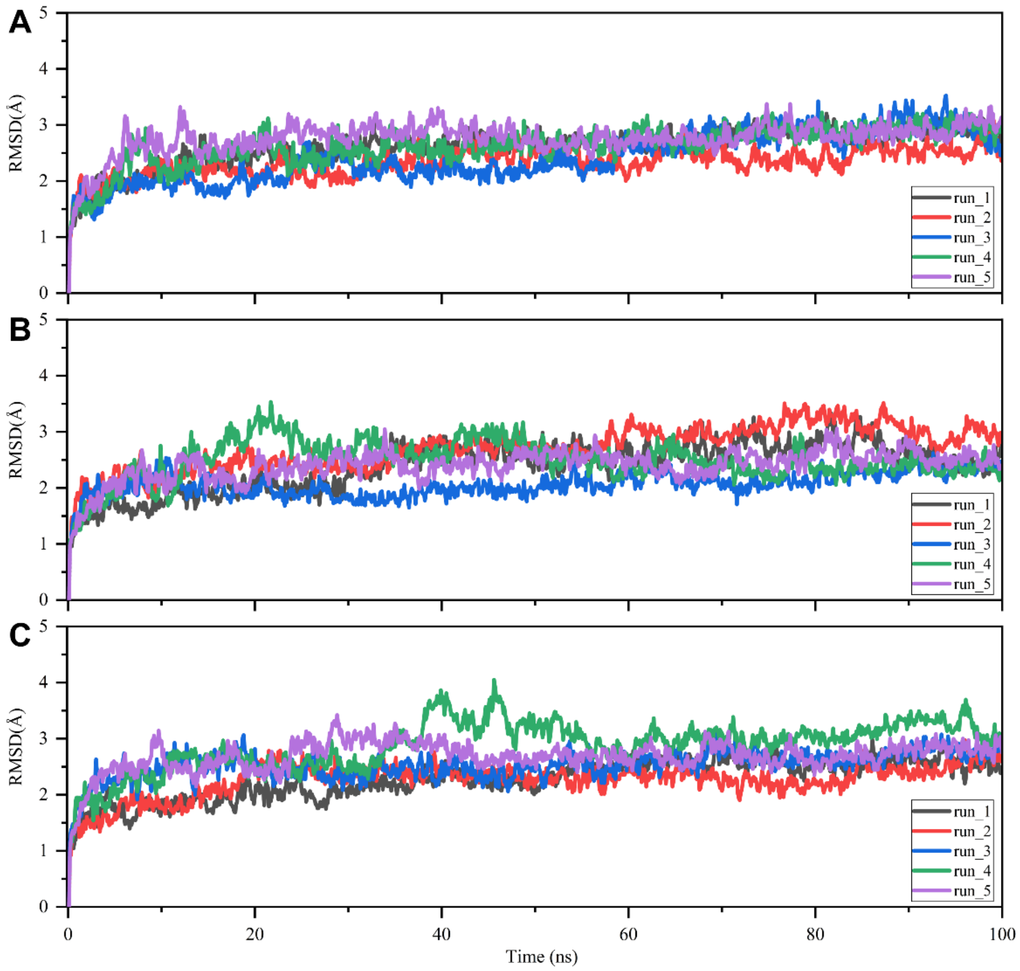

Figure S12. The root means square deviation (RMSD) values of wild type (A), branching GlcNAc (B) and bisecting GlcNAc (C) at Asn494 of P-gp

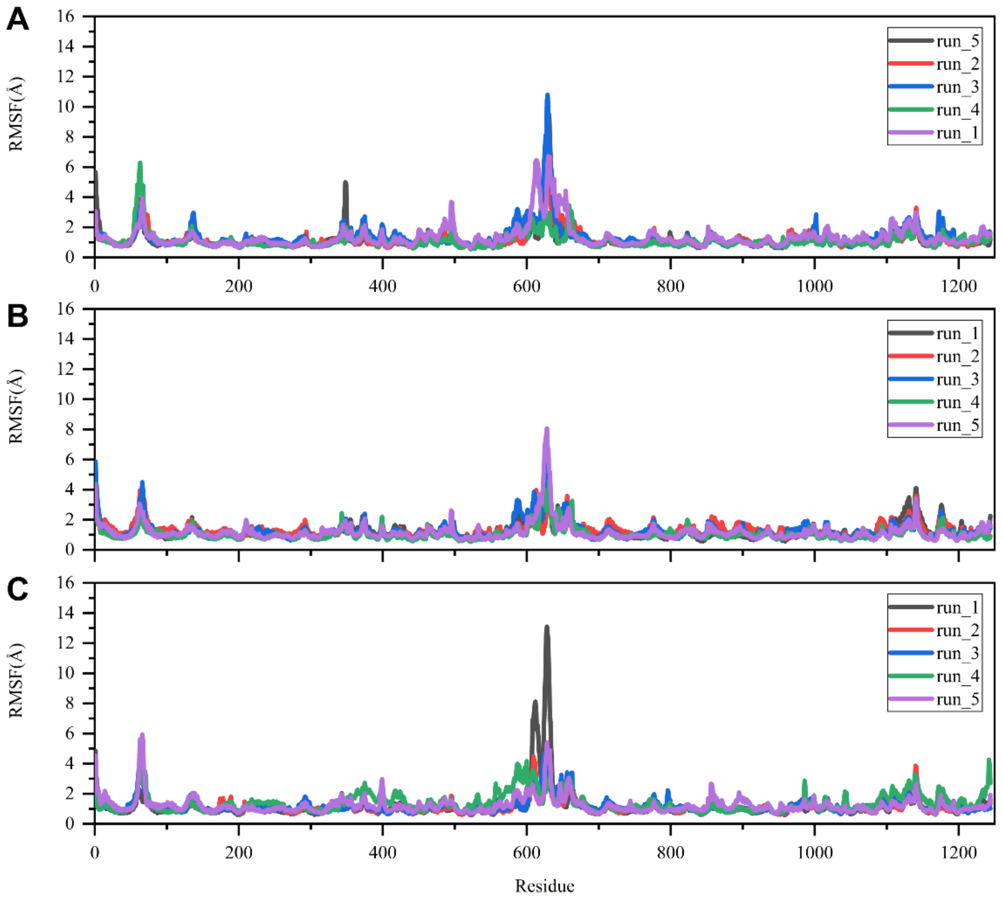

Figure S13. The root means square fluctuation (RMSF) values of wild type (A), branching GlcNAc (B) and bisecting GlcNAc (C) at Asn494 of P-gp

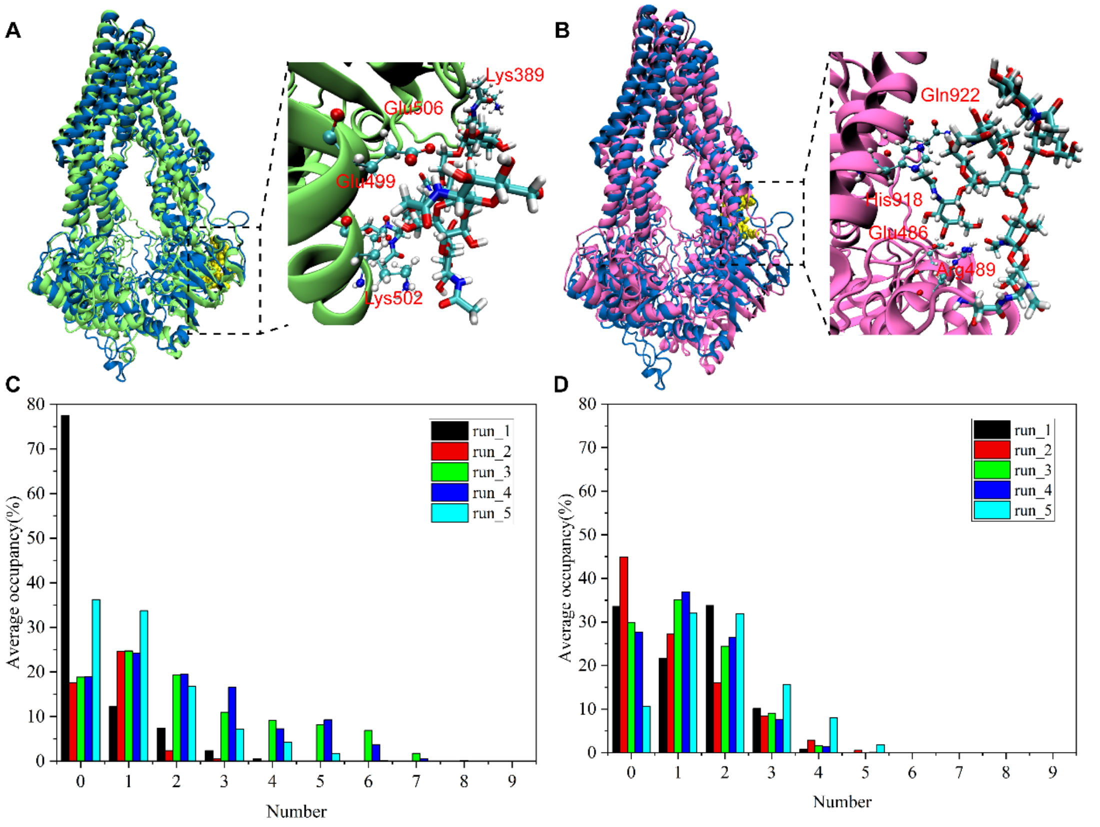

<u>Figure S14. Hydrogen bonding interactions driving the combination of P-gp and GlcNAc</u>

(A) The superposition of initial structures (indigo blue) and the final frame of Run1 (lime) in the presence of branching GlcNAc, with the details of binding sites displayed. (B) The structural characteristics of bisecting GlcNAc binding site on the surface of P-gp. The final frame of Run1 (mauve) is superposed to the initial structure of P-gp (indigo blue) with the binding site highlighted. (C) Hydrogen bonding occupancy between P-gp and branching GlcNAc. (D) Hydrogen bonding occupancy between P-gp and bisecting GlcNAc.

Table S1. Differentially expressed glycan-related genes in chemoresistant BC patients who attained RCB-III or imputed resistance to DOX

Table S2. Proteins identified to differentially interacted with MGAT3

Table S3. Intact glycopeptides decorated with bisecting GlcNAc

Table S4. Proteins identified to differentially interacted with P-gp

Table S5. Proteins identified in MCF7, $MCF7^{DOX}$ and $MCF7^{DOX}$/MGAT3 cells

Table S6. Proteins identified in MVs from MCF7, $MCF7^{DOX}$ and $MCF7^{DOX}$/MGAT3 cells

Table S7. Proximity biotinylated proteins associated with Annexin A1
